# Supplementary material for: Acetylation of histone H2B marks active enhancers and predicts CBP/p300 target genes
Source: Nat Genet. 2023 Apr 6;55(4):679–92. doi: 10.1038/s41588-023-01348-4 (PMC10101849; doi:10.1038/s41588-023-01348-4)
Supplement: Supplementary file 1 — Supplementary Notes 1 and 2, Figs. 1–18, Tables 1–3 and Source Data Fig. 1. [file 41588_2023_1348_MOESM1_ESM.pdf]

nature genetics

Article

https://doi.org/10.1038/s41588-023-01348-4

Acetylation of histone H2B marks active enhancers and predicts CBP/p300 target genes

In the format provided by the authors and unedited

Supplementary Information:

Supplemental Note 1:

For more than 2 decades, acetylation of H2BNT sites has been detected across diverse eukaryotes[59,60], even though the H2BNT sequence shows little sequence conservation between budding yeast, Drosophila, and human (Supplementary Fig. 18b-c). The current picture of H2BNTac genomic occupancy and regulation is confusing. In three different cell lines, H2BNTac sites showed varying correlations with each other and with other chromatin marks[9,22,23]. H2BNTac sites formed a 17-histone modification signature that marked virtually all active promoters[22]. Similar to H3K27ac and H3K4me3, promoter abundance of H2BK5ac was strongly correlated with global gene expression[42]. One study noted preferential enrichment of H2BK20ac at active enhancers and cell-type-specific promoters; but most notably, this study identified an entirely new class of enhancers that were exclusively marked with H2BK20ac and lacked the canonical enhancer mark H3K27ac[9]. Furthermore, H2BK20ac occupancy correlated with H2BK120ac, but not with other H2BNTac sites[9,23], suggesting that it is an outlier among H2BNTac marks. More recently, H2BK20ac was shown to be critical for recruiting histone macroH2A1, which then recruits PARP1 and CBP to promote the acetylation of H2BK12 and H2BK120[61].

The above-mentioned studies portrayed a complex picture of H2BNTac occupancy and left many mechanistic questions unresolved. For example, why is H2BK20ac an outlier among H2BNTac sites, and why do other H2BNTac sites lack enhancer specificity? How does the same acetyltransferase, CBP/p300, differentially acetylate H3K27 and H2BK20 in a locus-specific manner? Is H2BK20ac catalyzed by acetyltransferase other than CBP/p300 at the new class of H2BK20ac^{+}H3K27ac^{-} enhancers? Why does H2BK20ac genome occupancy resembles that of H2BK120ac, are these marks differentially regulated by acetyltransferase and deacetylases? The sequential H2BK20 and H2BK120 acetylation model[61] could explain why these marks co-occur, but it also suggests that H2BK20ac is already present before the recruitment macroH2A1-PARP1-CBP/p300, and H2BK12ac occur after that. What acetylates the initial H2BK20 before the recruitment of macroH2A1, and if PARP1-CBP acetylates H2BK12 and H2BK120 in the same regions[61]? If so, why do these two marks show dissimilar genomic occupancy[22,23]? How do H2BK20ac and other H2BNTac sites compare with other chromatin features in the prediction of enhancer target genes?

The lack of mechanistic understanding emerges as the primary reason for these discrepancies, which, combined with poor H2BNT sequence conservation, has hampered the adoption of H2BNTac as a reliable enhancer marker. Our prior mass spectrometry analyses clarified the site-specificity and dynamics of CBP/p300 targets[18]. We harnessed this knowledge to uncover and explain a unifying picture H2BNTac sites. This illustrates the usefulness of integrating proteomic and genomic analyses in obtaining an improved mechanistic understanding of chromatin marks.

Supplemental Note 2:

We cannot entirely rule out the possibility that antibodies used for establishing the H2BNTac signature may cross-react with other histone acetylation sites or recognize an off-target(s). But for the following reasons, we believe that the H2BNTac signature is robust and unlikely to arise from a lack of antibody specificity. (1) We used six different rabbit monoclonal antibodies, raised against 5 different H2BNTac sites, to establish the H2BNTac signature. (2) Histone termini are unstructured and antibody specificity is primarily dictated by linear sequences, not

3D conformation. Cross-reactivity can arise if amino acids flanking modified residues are identical. (3) Within the H2BNT, amino acids flanking lysine are highly dissimilar, making it unlikely that all monoclonal antibodies cross-react within H2BNT. (4) Guided by the sequence similarity, and the unique H2BNTac profile, we found one of the H2BK5ac antibodies as an outlier, explained its cross-reactivity with H3K27ac, and excluded it from our analyses. (5) Transcription inhibition preferentially increased H2BNTac in actively transcribed genes, consistent with the transcription-induced exchange of H2A-H2B[34,35]. (6) CBP/p300-dependent H2BNTac regulation in our ChIP-seq is consistent with mass spectrometry-based analyses of H2BNTac sites[18]. The only other histone sites that show similarly strong, global downregulation after CBP/p300 inhibition are a subset of sites present in histone H3 N-terminus, whose genome occupancy profiles are dissimilar from H2BNTac. (7) CREs marked with H2BNTac bear all the canonical features of active enhancers, including DHS, H3K4me1, H3K27ac, and MED1 binding. (8) H2BNTac marked regions show a high validation rate in orthogonal enhancer activity assays.

Supplementary Fig. 1

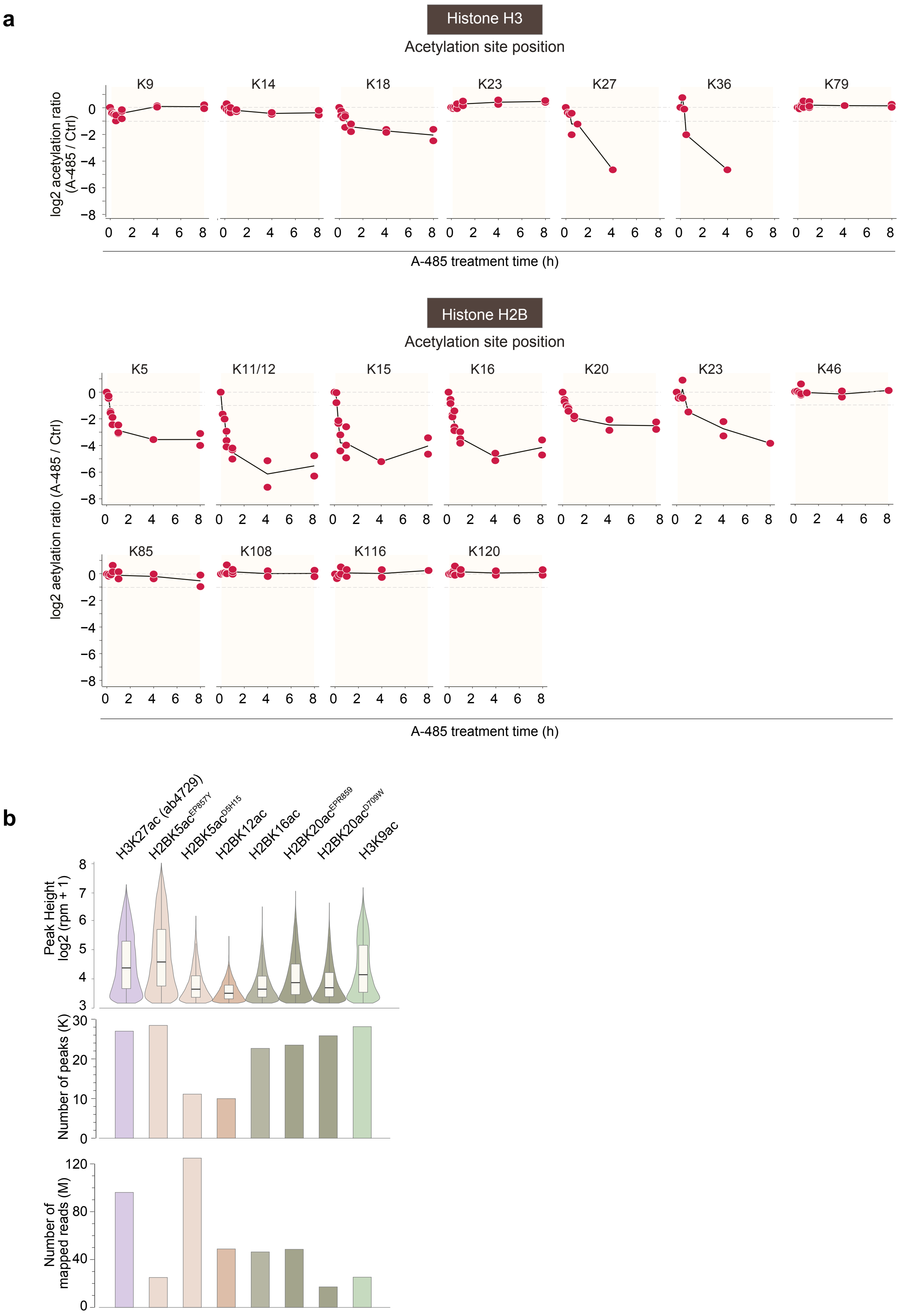

Supplemental Fig. 1. CBP/p300-dependent acetylation of H3K27ac and H2BNTac, and a summary of ChIP-seq analyses. a, Deacetylation kinetics of histone H3 and H2B sites after CBP/p300 inhibition in mouse embryonic fibroblasts. Histone H3 and H2B acetylation site time-course data were re-analyzed from Weinert et al.[18]. **b,** Summary of ChIP-seq analyses. The upper panel shows a comparison of H3K27ac, H3K9ac, and H2BNTac site ChIP signal intensity, the middle panel shows the number of identified peaks, and the lower panel shows the number of mapped read counts. The box plots display the median, upper and lower quartiles, and whiskers show 1.5× interquartile range (IQR). Note that the number of identified peaks does not directly scale with the number of mapped reads. Instead, the number of identified peaks is better reflected in peak height; H2BK5ac^{D5H15} and H2BK12ac show the lowest peak height and the lowest number of identified peaks. Lower H2BK5ac^{D5H15} and H2BK12ac peak height may reflect weaker enrichment efficiency of the used antibodies and/or lower abundance of these marks.

Supplementary Fig. 2

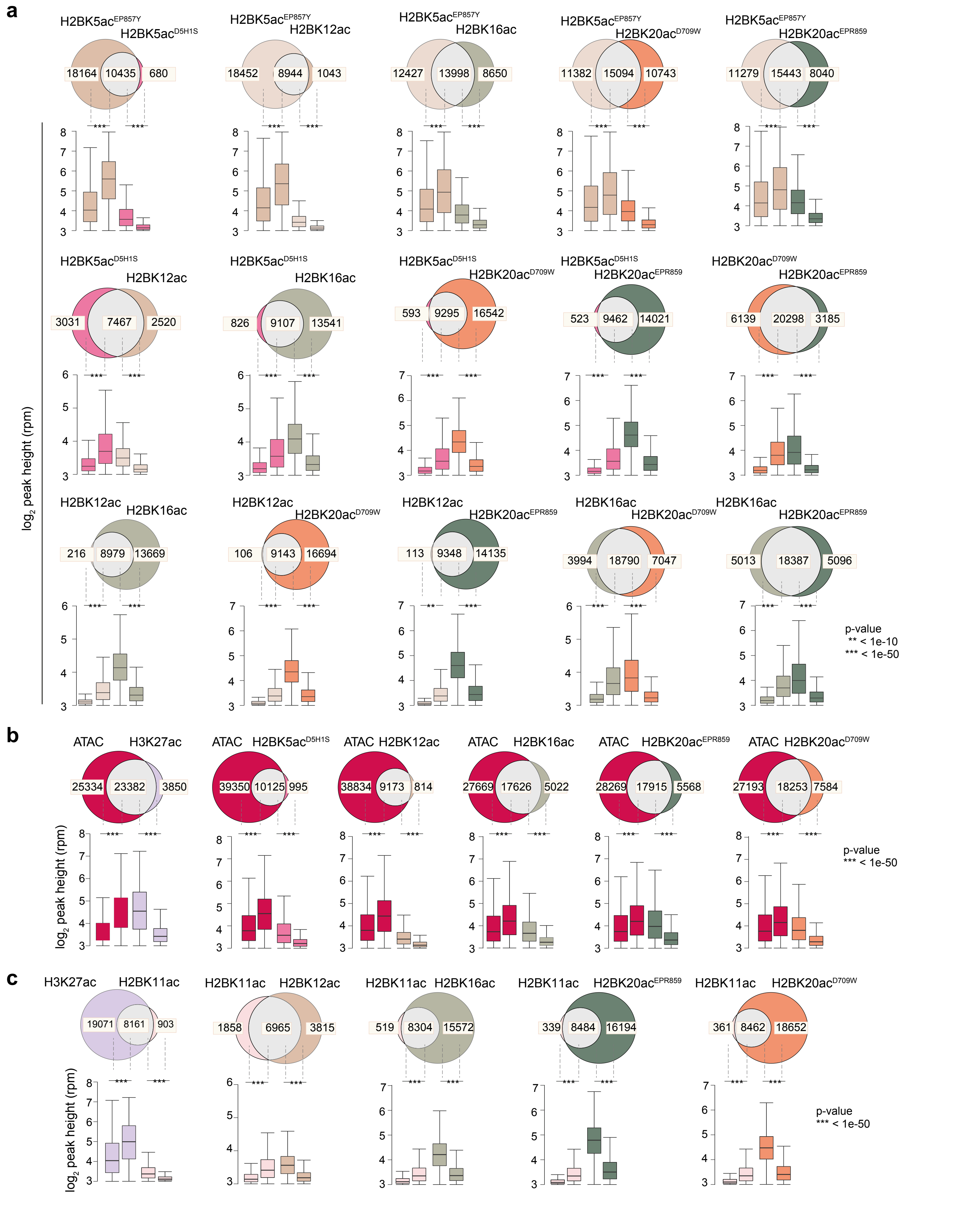

Supplemental Fig. 2. H2BNTac sites occupy the same genomic regions. a, Venn diagrams show the overlap of ChIP-seq peaks between the indicated H2BNTac marks. Below each Venn diagram, box plots show the distribution of peak height for the overlapping and non-overlapping peaks for each of the compared acetylation marks. Due to difficulty in peak calling and variation in peak width, a peak in one dataset can overlap with more than one peaks in another dataset or vice versa, which leads to a slightly different number of overlapping peaks between H2BNTac peaks. In this case, a higher peak number is shown as a representative overlapping peak number. **b,** Overlap of H3K27ac and H2BNTac peaks with ATAC-seq peaks in mESC. Bar charts, below Venn diagrams, show the peak height of the indicated histone marks or ATAC-seq. **c,** H2BK11ac overlaps with other H2BNTac sites. Shown is the overlap of H2BK11ac^{+} regions with the indicated H3K27ac and H2BNTac marks. The number of ChIP-seq biological replicates (a-c): H2BK5ac^{D5H1S} (n = 4), H3K27ac (n = 3), H2BK12ac (n = 2), H2BK16ac (n = 2), H2BK20ac^{EPR859} (n = 2), H2BK11ac (n = 1), H2BK20ac^{D709W} (n = 1), H3K9ac (n = 1). As a note, the H2BK11ac antibody became available after most of the other experiments and analyses were completed. Because of this late addition of these data and limited coverage of H2B acetylated regions by this antibody, H2BK11ac was not included in other comparisons. The box plots display the median, upper and lower quartiles, and whiskers show 1.5× interquartile range (IQR). Two-sided Mann–Whitney U test, adjusted for multiple comparisons by Benjamini and Hochberg method; **P < 1e-10, ***P < 1e-50.

Supplementary Fig. 3

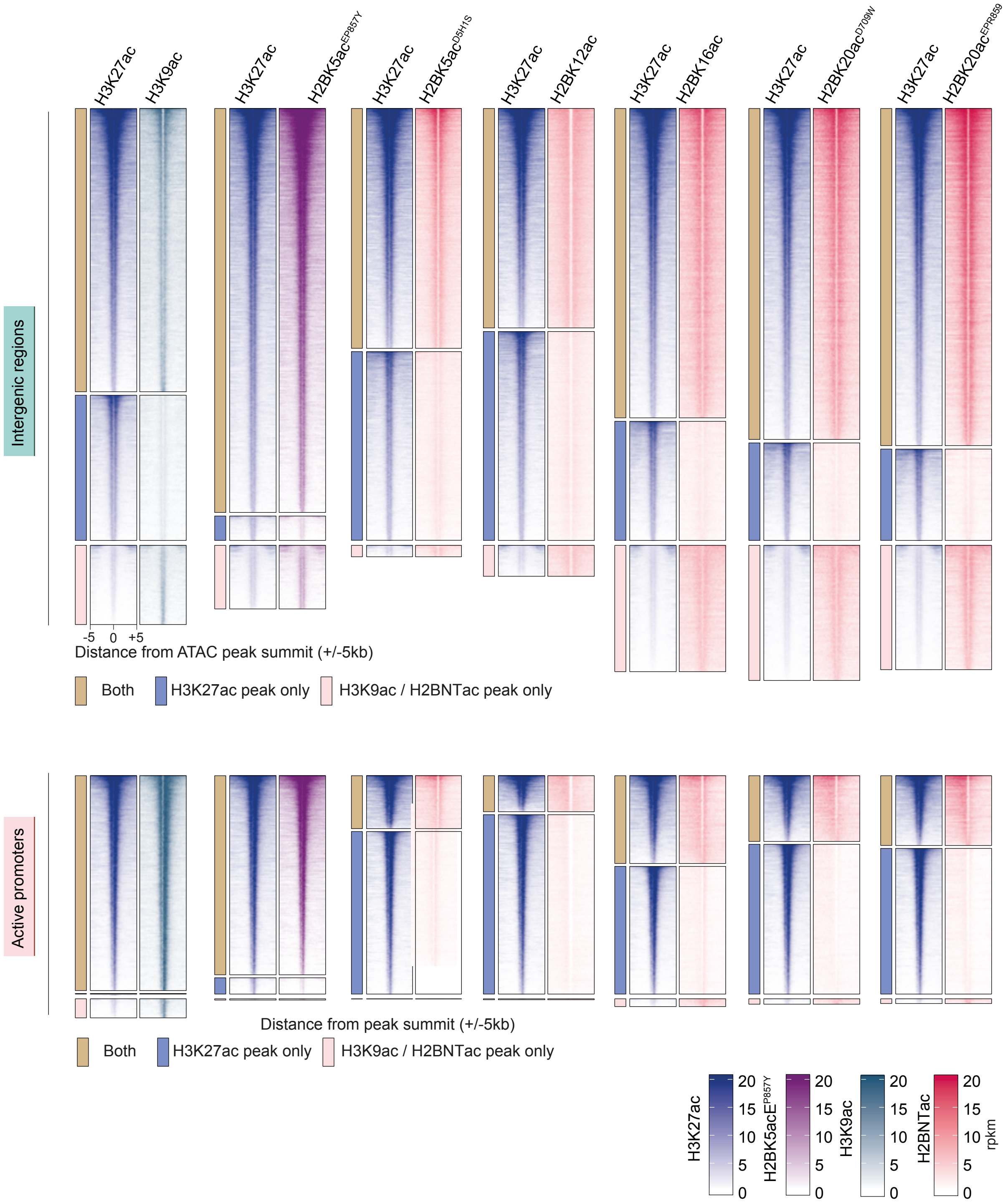

Supplemental Fig. 3. Distinct genomic occupancy of H3K27ac, H3K9ac, and H2BNTac in promoters and intergenic regions. Heatmaps of H3K27ac, H3K9ac, and the indicated H2BNTac mark peak profiles (within +/- 5kb from TSS) in mESC. Peaks are centered around the ATAC-seq peak. H3K27ac-positive peaks are ordered based on H3K27ac intensity. The upper panels show peaks localizing to the intergenic regions, and the lower panels show peaks mapping to the promoters of actively transcribed genes.

Supplementary Fig. 4

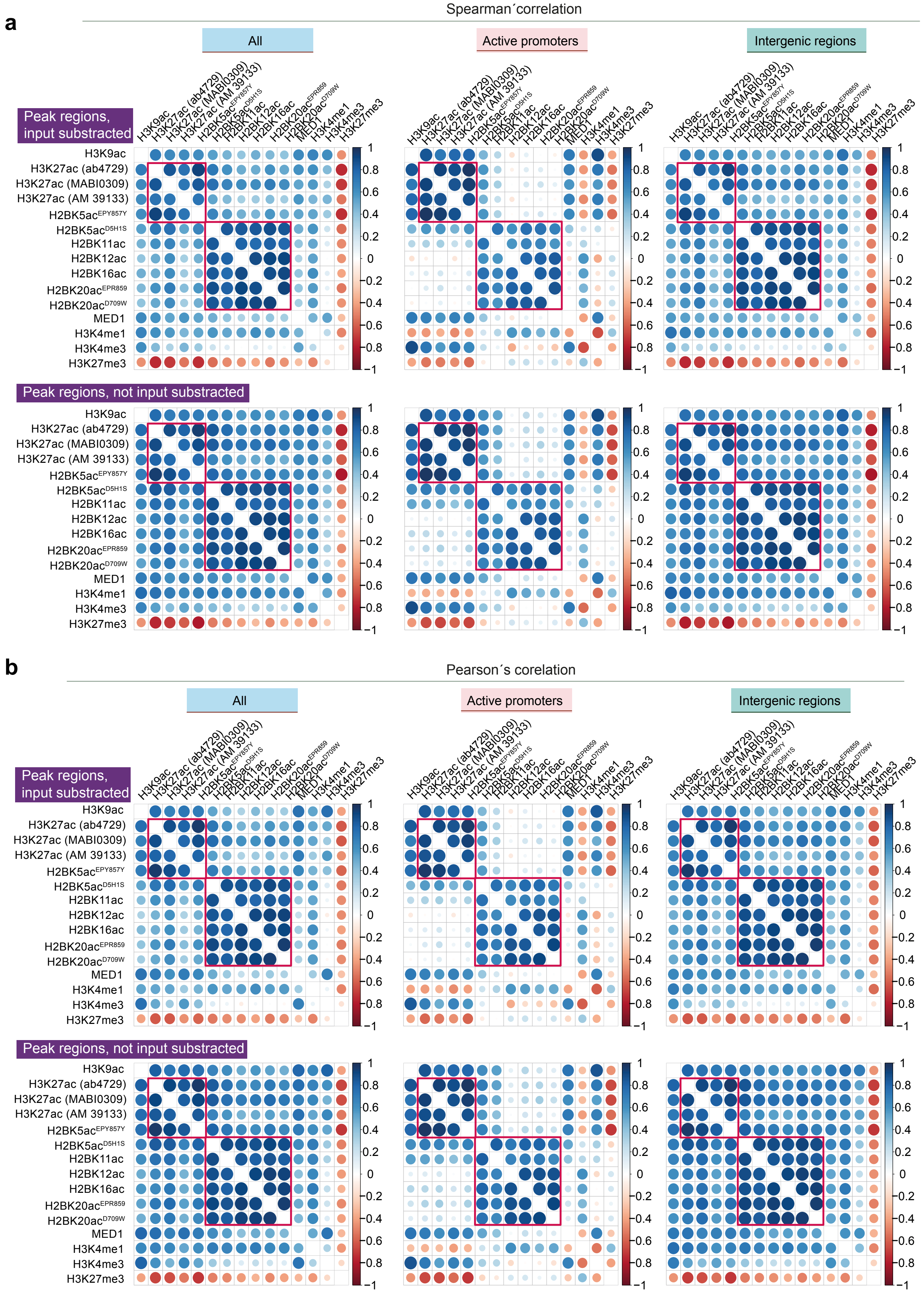

Supplemental Fig. 4. H3K27ac and H2BNTac distinctly correlate with other chromatin marks. a-b, Genome-wide correlation among the indicated chromatin marks. Correlations (Spearman´s ρ, panel a; and Pearson´s r, panel b) are calculated between the indicated marks using a 2kb window in peak regions. H2BNTac strongly correlates with each other and shows a variable positive correlation with H3K4me1, H3K27ac, H3K9ac, and MED1. Notably, H2BNTac very poorly correlates with H3K4me3 and negatively correlates with H3K27me3.

Supplementary Fig. 5

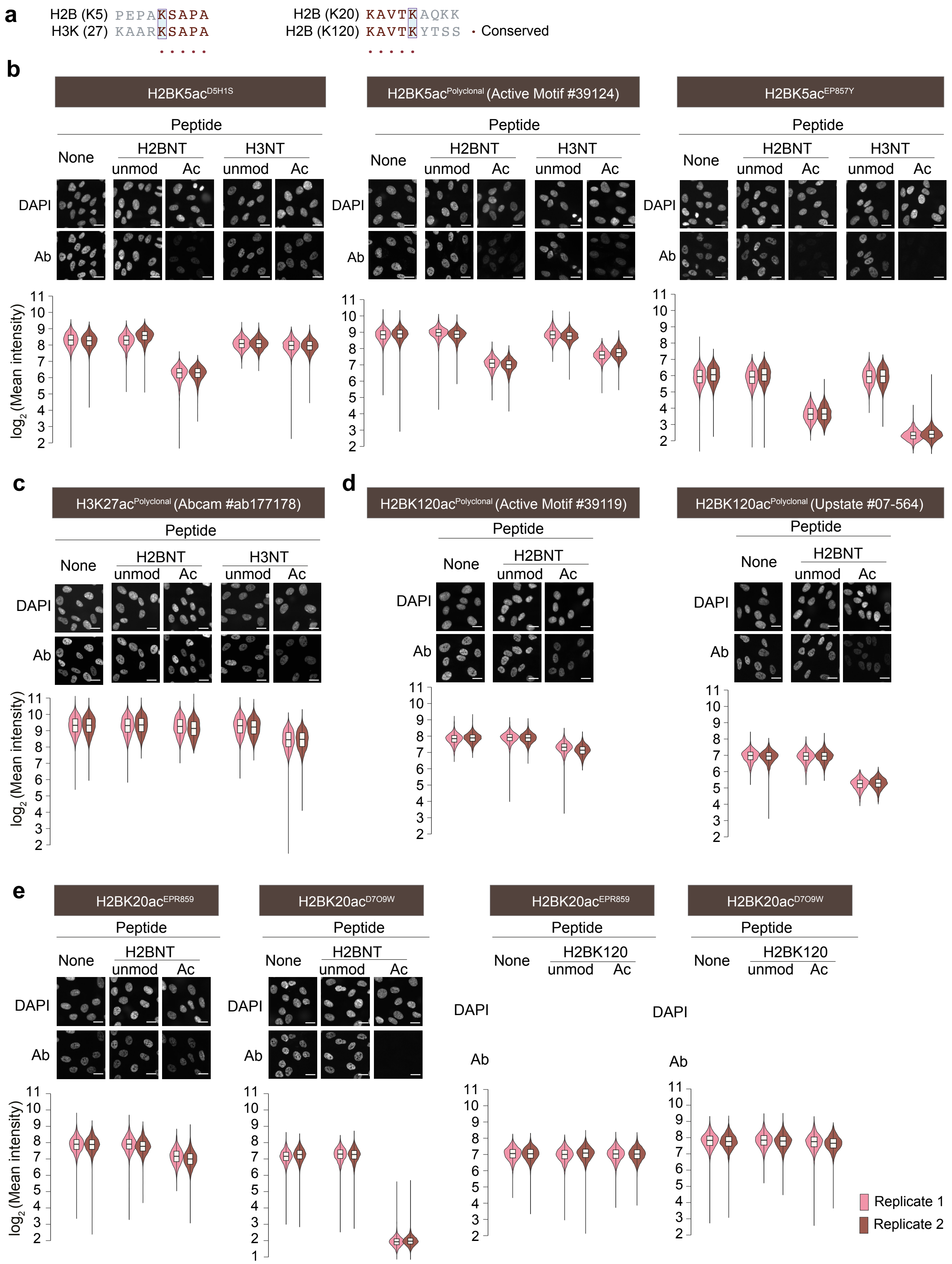

Supplemental Fig. 5. Previously used H2BK5ac and H2BK120ac ChIP antibodies cross-react with other histone acetylation sites. a, The sequence similarity of amino acids flanking H2BK5 and H3K27, and H2BK20 and H2BK120. Acetylated lysine is positioned in the middle and marked with a box. Identical amino acids are marked with dots. **b-e,** Analysis of H2BK5ac (b), H3K27ac (c), H2BK120ac (d), and H2BK20ac (e) antibody specificities. Cells were immunostained with the stated antibodies in the absence, or presence of the indicated unmodified (Unmod) or acetylated (Ac) peptides. Shown are the representative immunofluorescence images. Scale bar indicates 20µm. Violin plots show the distribution of immunofluorescence signals for the indicated antibodies and treatment conditions. For each condition, 3,000 cells were quantified. Data are from 2 independent biological replicates. The box plots show the median, interquartile range (IQR), and whiskers show 1.5x IQR. Unmod (unmodified peptide), Ac (acetylated peptide), Ab (antibody), H2BNT (H2B N-terminus peptide), and H3NT (H3 N-terminus peptide). Peptide sequences used for antibody specificity testing are provided in Supplemental Table 2.

Supplementary Fig. 6

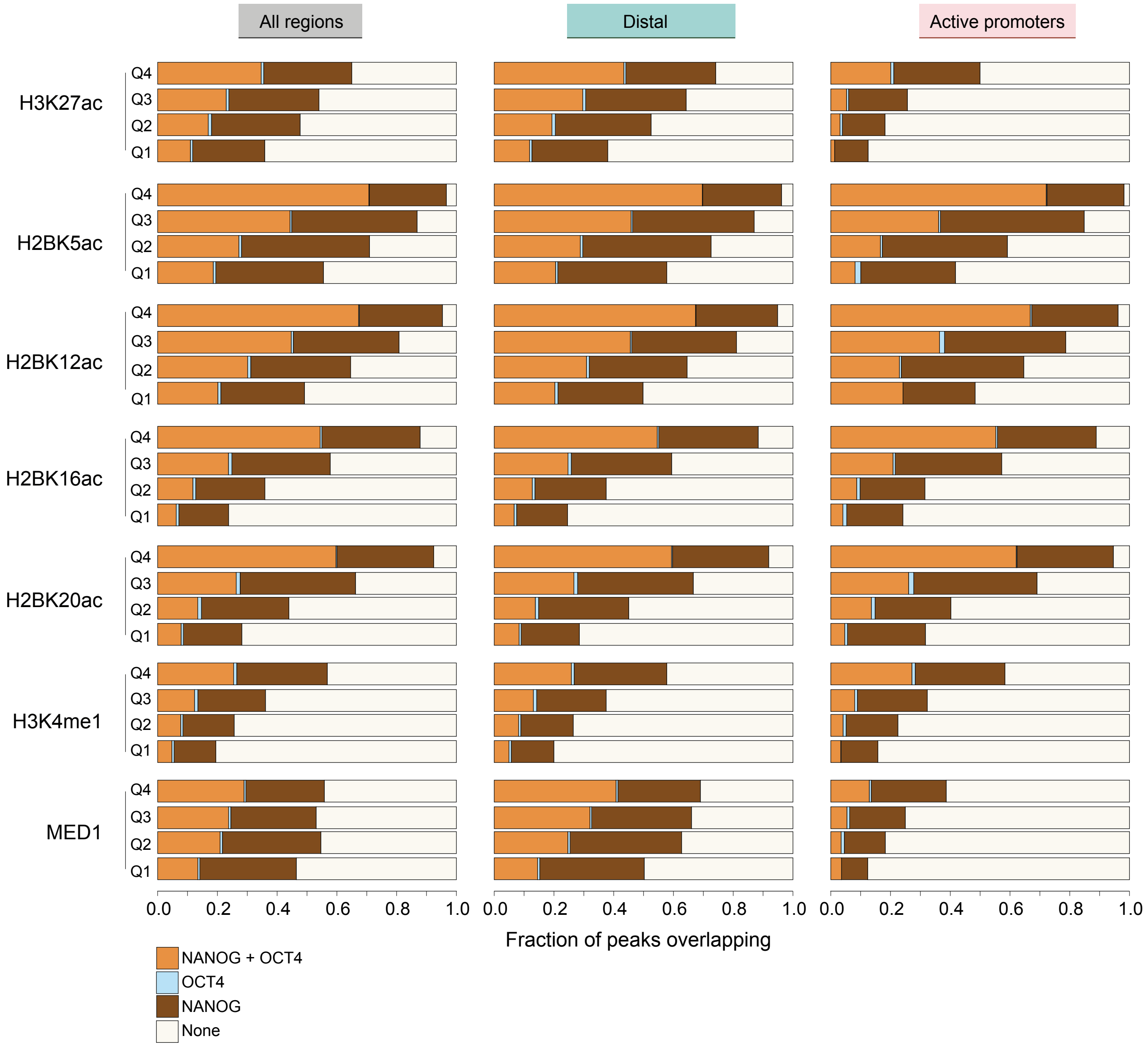

Supplemental Fig. 6. H2BNTac[+] regions are occupied by NANOG and OCT4. Bar charts showing the fraction of H2BNTac, H3K27ac, H3K4me1, and MED1 regions bound by NANOG and/or OCT4. Based on ChIP signal intensity, H2BNTac, H3K27ac, H3K4me1, and MED1 regions were grouped into quartiles, and within each group, the fraction of regions overlapping with NANOG and/or OCT4 is shown. Co-occupancy of the indicated chromatin marks and OCT4 and/or NANOG was analyzed in the indicated genomic regions. All, all peaks; Active promoter, peaks mapping to ±1kb of active promoters (TPM ≥2 and marked with H3K4me3); Intergenic, peaks occurring outside promoter regions.

Supplementary Fig. 7

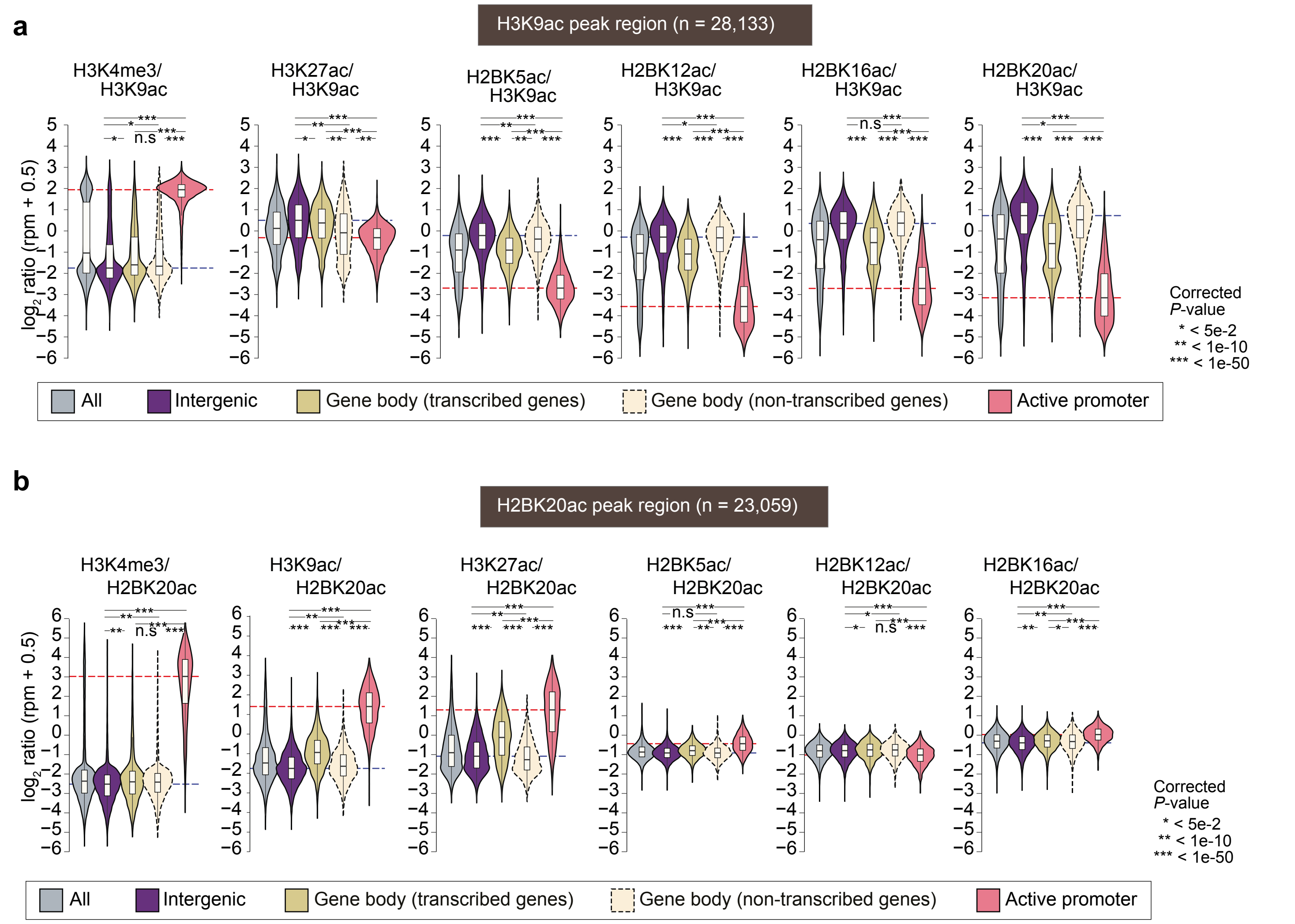

Supplemental Fig. 7. H2BNTac separates candidate enhancers and active promoters. a-b, Shown are the relative ChIP-seq signal intensities of the indicated chromatin marks at H3K9ac occupied regions (a) or H2BK20ac regions (b) in mESC. Active promoters (±1 kb from TSS) are defined as promoters of actively transcribed genes in mESC (TPM ≥2) and marked with H3K4me3. ChIP-seq peaks are grouped into the following categories: All, all peaks; Intergenic, peaks occurring outside promoters and gene body; Gene body (transcribed genes), peaks occurring within transcribed gene body; Gene body (non-transcribed genes), peaks occurring within the non-transcribed gene body. The box plots display the median, upper and lower quartiles, and whiskers show 1.5× interquartile range (IQR). The dotted lines indicate the median ratio of the indicated chromatin marks at intergenic regions (blue) and active promoters (red). The number of biological replicates: H2BK5ac^{D5H1S} (n = 4), H3K27ac (n = 3), H2BK20ac (n = 3), H2BK12ac (n = 2), H2BK16ac (n = 2), H3K4me3 (n = 1), H3K9ac (n = 1). The box plots display the median, upper and lower quartiles, and whiskers show 1.5× interquartile range (IQR). Two-sided Mann–Whitney U test, adjusted for multiple comparisons by Benjamini and Hochberg method; n.s, not significant $P \geq 0.05$, *$P < 0.05$, **$P <$ 1e-10, ***$P <$ 1e-50.

Supplementary Fig. 8

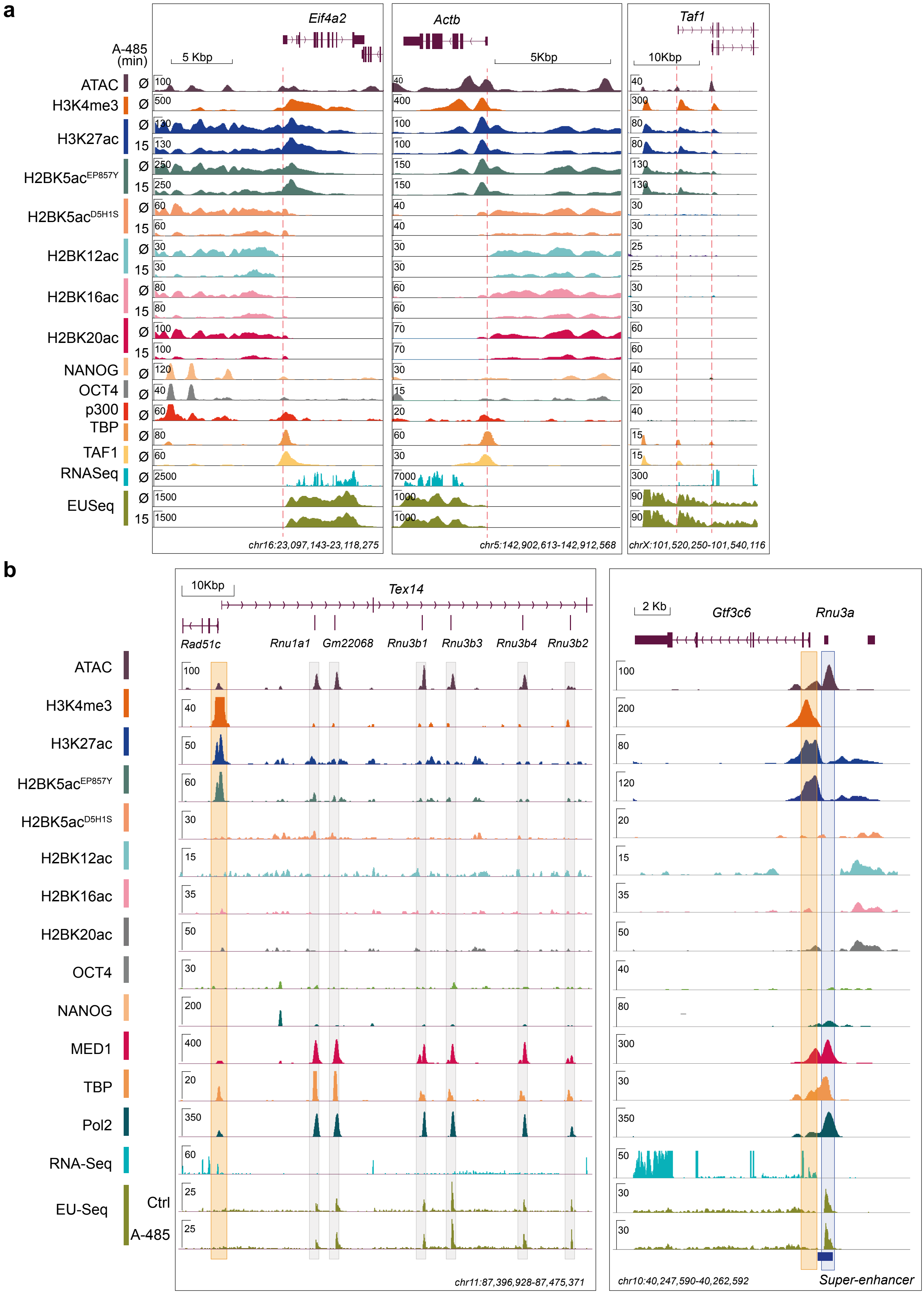

Supplemental Fig. 8. H2BNTac distinguishes candidate enhancers from protein-coding and snoRNA-coding gene promoters. a, H2BNTac distinguishes candidate enhancers from proximally occurring promoters of the housekeeping genes *Actb* and *Eif4a2*. The dotted line demarcates intergenic H2BK20ac⁺ candidate enhancer regions from the gene promoters. *Taf1* upstream H3K27ac⁺ peak lacks H2BNTac but is marked with H3K4me3, identifying it as a putative promoter. This is consistent with the EU-seq signal in this region. The dotted line demarcates intergenic and the currently annotated *Taf1* TSS. **b,** A genome browser view of *Rad51c* and *Tex14* proximal (left) and *Gtf3c6* proximal (right) regions. *Rad51c*, *Tex14*, and *Gtf3c6* promoters are shadowed in orange, and *Gtf3c6* upstream candidate super-enhancer[62] and the six regions that were previously interpreted as *Rad51c*-proximal enhancers[29], are shadowed in light blue and light grey, respectively. The candidate enhancers, indicated by shadowed boxes, are marked with a high level of ATAC-seq, MED1, TBP, and Pol2, but have little undetectable H3K4me3 and H2BNTac. While *Rad51c*, *Tex14*, and *Gtf3c6* expression are detected in RNA-seq, the candidate enhancer regions lack a detectable RNA-seq signal. Instead, they show a very high level of nascent RNA transcription, which remains unaffected by the A-485 treatment. As indicated, a closer inspection of the expanded genome annotation reveals all seven regions as snoRNA genes.

Supplementary Fig. 9

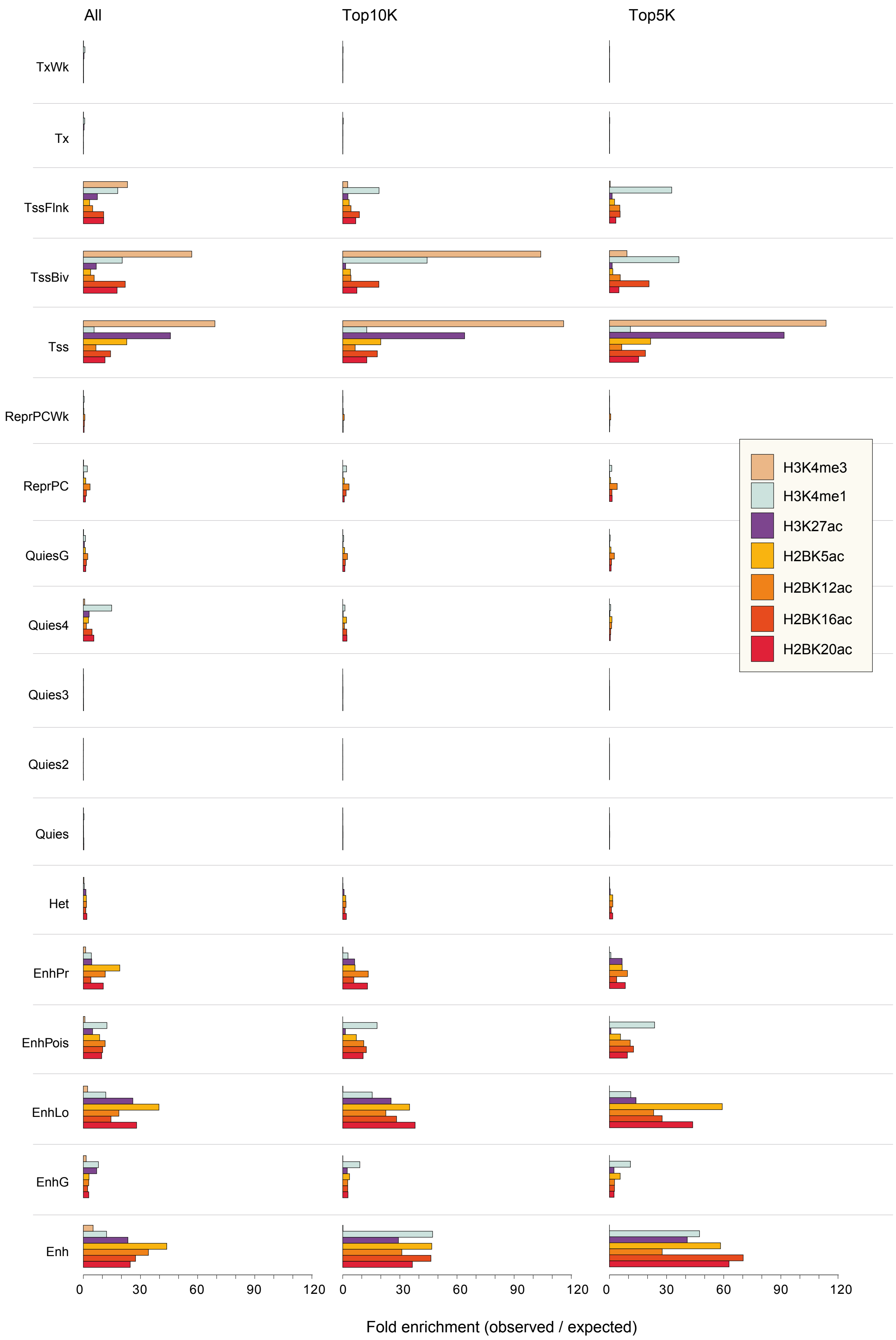

Supplemental Fig. 9. Enrichment of H2BNTac in ChromHMM defined chromatin states in mESC. Shown is the enrichment of the specified histone marks in the indicated ChromHMM-defined chromatin states. The top five thousand (Top 5K) and top ten thousand (Top10K) peaks were chosen based on the peak heights of the specified chromatin marks. mESC ChromHMM annotations were obtained from van der Velde et al.[63].

Supplementary Fig. 10

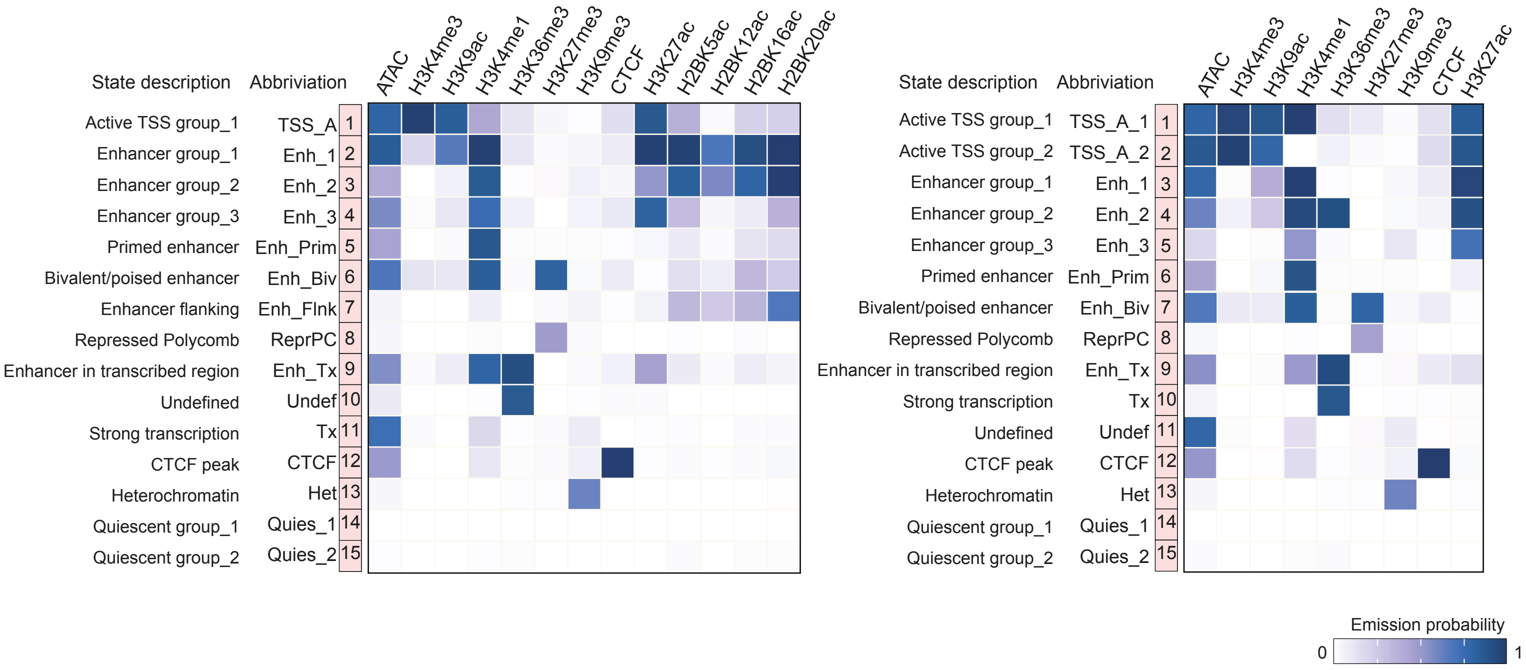

Supplemental Fig. 10. ChromHMM-based prediction of mESC chromatin states, with or without H2BNTac. Shown are the ChromHMM states in mESC, predicted using a combination of chromatin marks, with or without including H2BNTac. ChromHMM states were generated using a 15-state model, either based on nine different chromatin features (ATAC, CTCF, H3K4me3, H3K4me1, H3K36me3, H3K9ac, H3K9me3, H3K27me3, and H3K27ac) (left panel), or by additionally including the indicated H2BNTac marks (right panel). The heatmaps show the emission parameters in which each row corresponds to a different state, and each column corresponds to a distinct chromatin mark. The identity of chromatin marks is indicated at the top of the heatmaps. The color represents the probability of observing the mark in the state; the darker color represents a greater probability. ChromHMM states were annotated manually. Descriptions for the annotated states, followed by a state abbreviation, are shown to the right of the heatmaps.

Supplementary Fig. 11

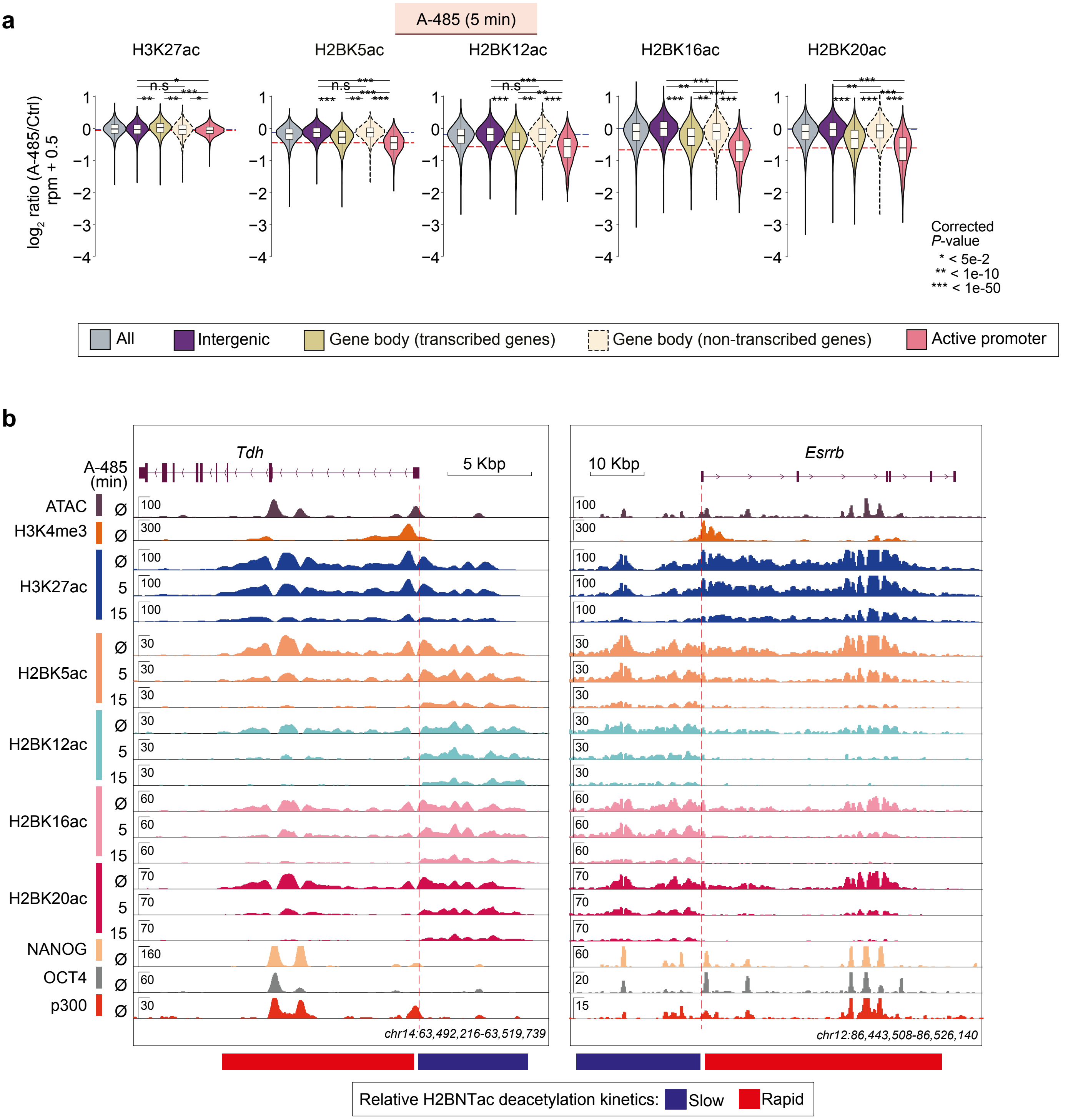

Supplemental Fig. 11. H2BK20ac is rapidly removed in actively transcribed promoters and gene body regions. a, CBP/p300 inhibition causes H2BNTac deacetylation within 5 minutes. Shown the fold-change in H3K27ac and H2BNTac site ChIP signal in untreated and A-485 treated (5 min) cells. Different genomic regions were classified as defined in Extended Data Fig. 7. Dotted lines indicate the median ratio at intergenic and active promoter regions. The box plots display the median, upper and lower quartiles, and whiskers show 1.5× interquartile range (IQR). The number of ChIP-seq biological replicates: H2BK5ac (n = 2), H3K27ac (n = 2), H2BK12ac (n = 1), H2BK16ac (n = 1), H2BK20ac (n = 1). Two-sided Mann–Whitney U test, adjusted for multiple comparisons by Benjamini and Hochberg method; n.s, not significant $P \geq 0.05$, *$P < 0.05$, **$P <$ 1e-10, ***$P <$ 1e-50. **b,** Genome browser view of *Tdh* and *Essrb* loci showing rapid removal of H2BNTac in actively transcribed gene body regions after CBP/p300 inhibition. As compared to intergenic regions, H2BNTac is more rapidly downregulated in enhancers occurring within transcribed gene body regions. In contrast, H3K27ac is similarly decreased at active promoters and candidate enhancers located in intergenic or gene body regions. The dotted lines demarcate intergenic regions (blue) and active promoters (red).

Supplementary Fig. 12

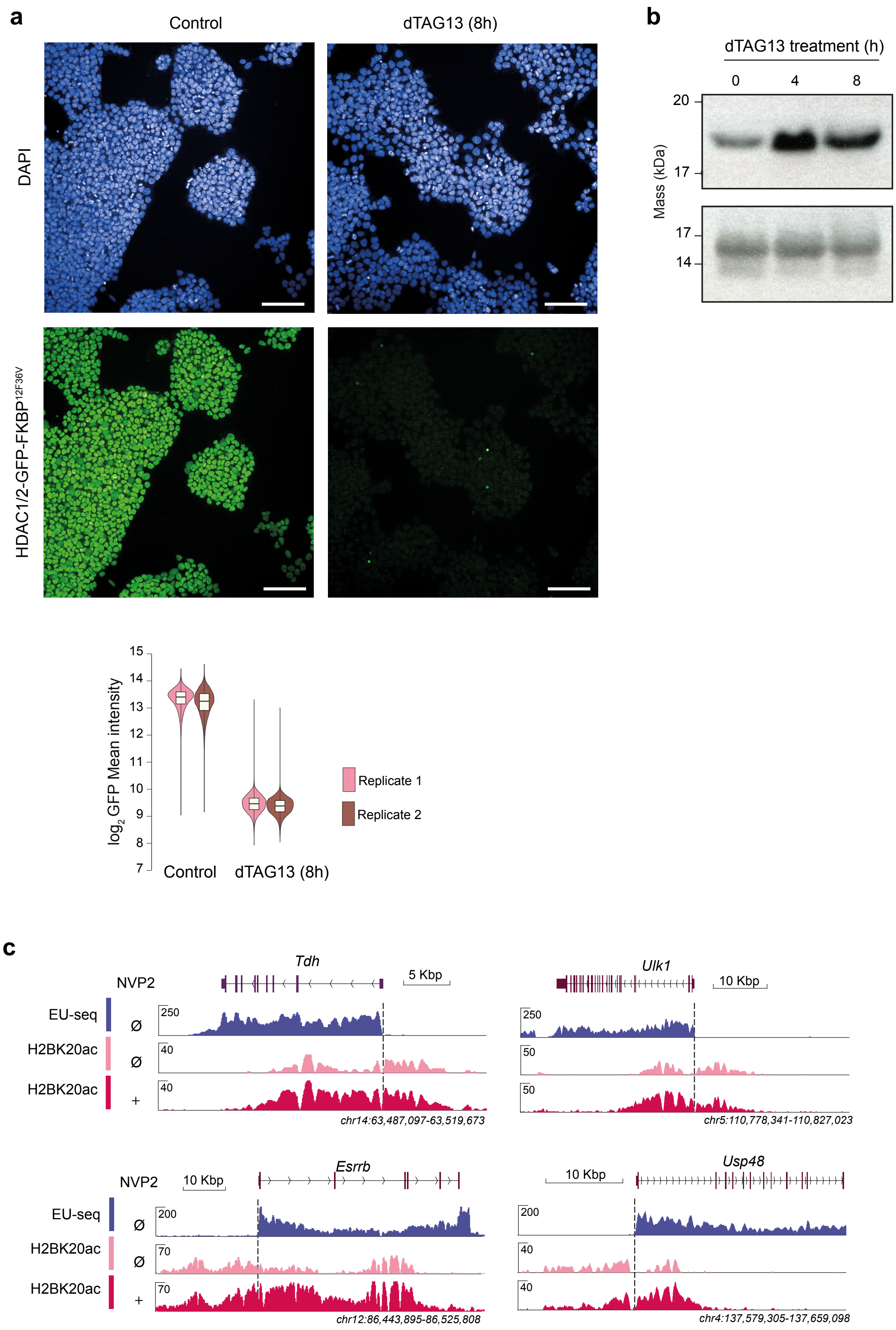

Supplemental Fig. 12. HDAC1/2 deacetylates H2BNTac, and transcription inhibition increases H2BNTac. a, Endogenous HDAC1/2 were fused with GFP-FKBP12^{F36V} and acutely depleted by treating cells with dTAG13 (8h). Depletion of HDAC1/2-GFP-FKBP12^{F36V} was confirmed by analyzing GFP expression by microscopy. The representative images show loss of GFP signal in dTAG13 treated HDAC1/2-GFP-FKBP12^{F36V} cells. Scale bar indicates 10µm. Violin plots show the distribution of GFP signals for the indicated treatment conditions. For each condition, >29,000 cells were quantified. Data are from 2 independent biological replicates. The box plots show the median, interquartile range (IQR), and whiskers show 1.5x IQR. **b,** Immunoblot showing acetylation of H2BK20ac in control and HDAC1/2 depleted mESC (n=2). The same lysates were run on a separate gel and immunoblotted for total histone H3. **c,** Representative genome browser tracks showing transcription inhibitor (NVP-2)-induced preferential increase in H2BK20ac in actively transcribed regions. H2BK20ac ChIP-seq was performed in mESC treated without or with NVP-2 (2h). The dotted line demarcates intergenic actively transcribed regions from upstream intergenic regions.

Supplementary Fig. 13

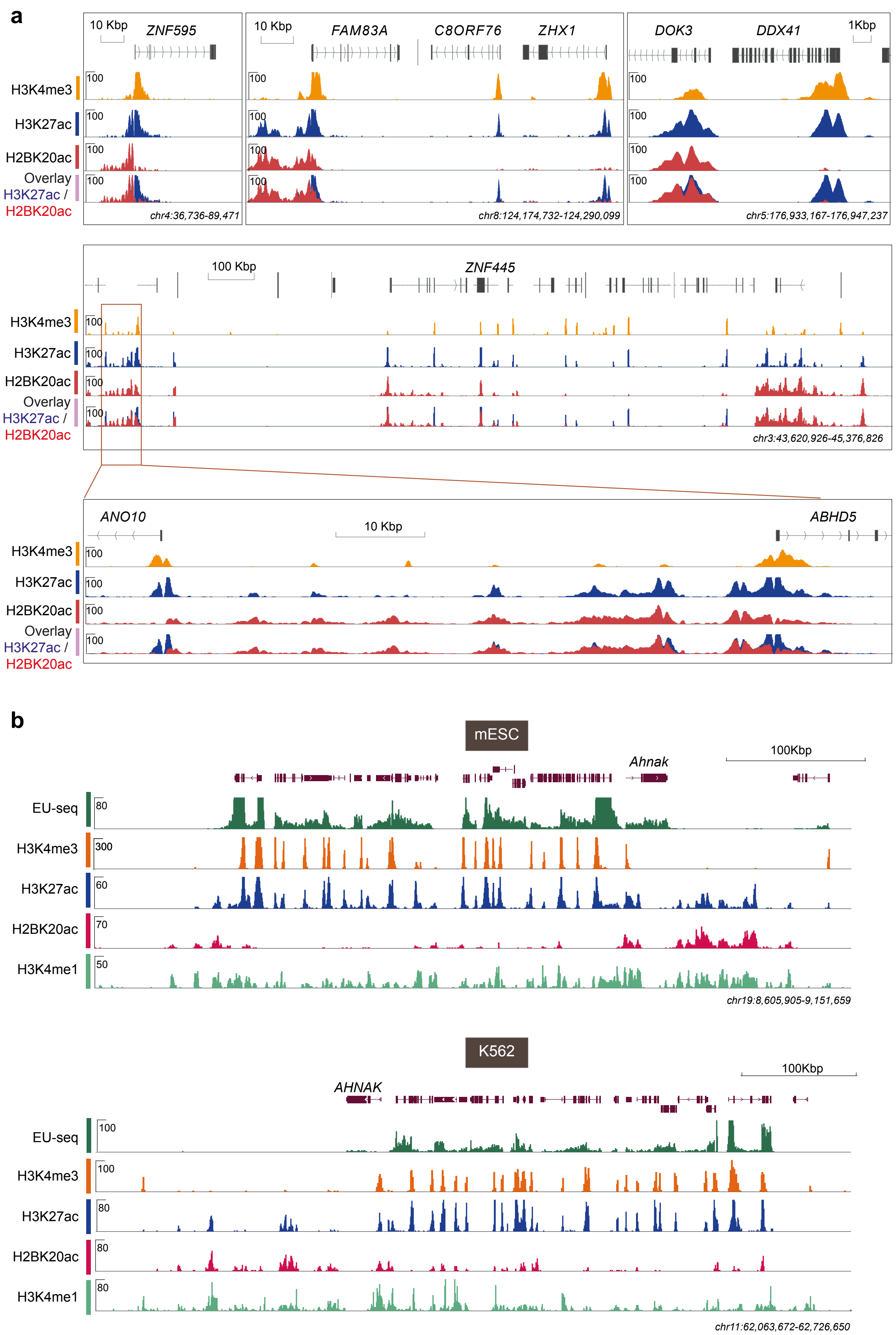

Supplemental Fig. 13. H2BK20ac complements other histone marks in distinguishing candidate enhancers from active promoters. a, Genome browser view of representative regions showing distinct occupancy profiles of H3K4me3, H3K27ac, and H2BK20ac in K562 cells. Note that H2BK20ac and H3K27ac show distinct enrichment in promoters. Some promoters are marked by both H2BK20ac and H3K27ac, whereas others are only marked by H3K27ac. Distal regions, marked with low H3K4me3, are similarly marked by H2BK20ac and H3K27ac. **b,** Representative genomic regions in mESC (upper panel) and K562 (lower panel) showing different occupancy patterns of H3K27ac, H2BK20ac, H3K4me3, and H3K4me1 in promoters and candidate enhancer regions. Most of the active promoters, marked with a high level of H3K27ac and H3K4me3, show a low level of H2BK20ac. Candidate enhancers, near the *Ahnak* and *AHNAK* regions, display low H3K4me3 and are co-marked with a similar level of H2BK20ac and H3K27ac.

Supplementary Fig. 14

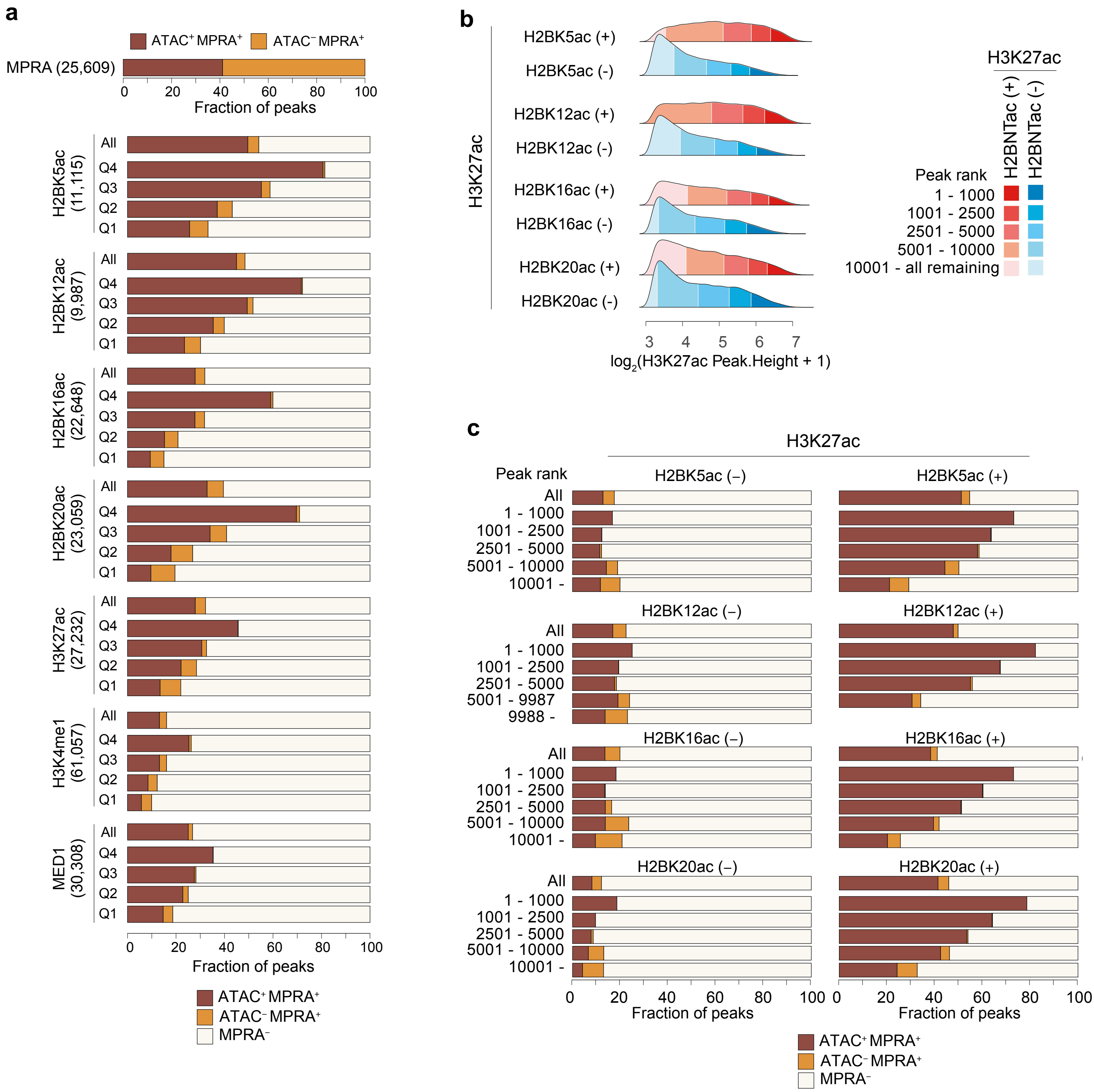

Supplemental Fig. 14. A high proportion of H3K27ac⁺H2BNTac⁺ peaks score positively in MPRA assay. a, Shown is the overlap of H2BNTac, H3K27ac, H3K4me1, and MED1 regions with ATAC⁺MPRA⁺ or ATAC⁻MPRA⁺ regions in mESC. The indicated chromatin marks were grouped into quartiles (Q4-Q1) based on ChIP-seq signal intensity, and the overlap with MPRA regions is shown within each quartile as well as for all peaks (All). **b,** Based on the co-occurrence of the indicated H2BNTac marks, H3K27ac peaks were grouped into two groups: H3K27ac⁺H2BNTac⁺ and H3K27ac⁺H2BNTac⁻. Within each group, peaks were ranked based on H3K27ac peak height. Shown are the density plots of H3K27ac⁺H2BNTac⁺ and H3K27ac⁺H2BNTac⁻ ChIP-seq peak height in the indicated rank categories. **c,** H3K27ac peaks in mESC were grouped into two groups, H3K27ac⁺H2BNTac⁺ and H3K27ac⁺H2BNTac⁻, and ranked as defined in panel (a). In the specified rank categories, the fraction of peaks overlapping with indicated groups of MPRA-defined candidate enhancers are shown. Note that a greater fraction of H3K27ac⁺H2BNTac⁺ peaks overlap with MPRA⁺ candidate enhancers than H3K27ac⁺H2BNTac⁻ peaks. A majority of ATAC⁺MPRA⁺ regions are marked by H2BNTac and/or H3K27ac.

Supplementary Fig. 15

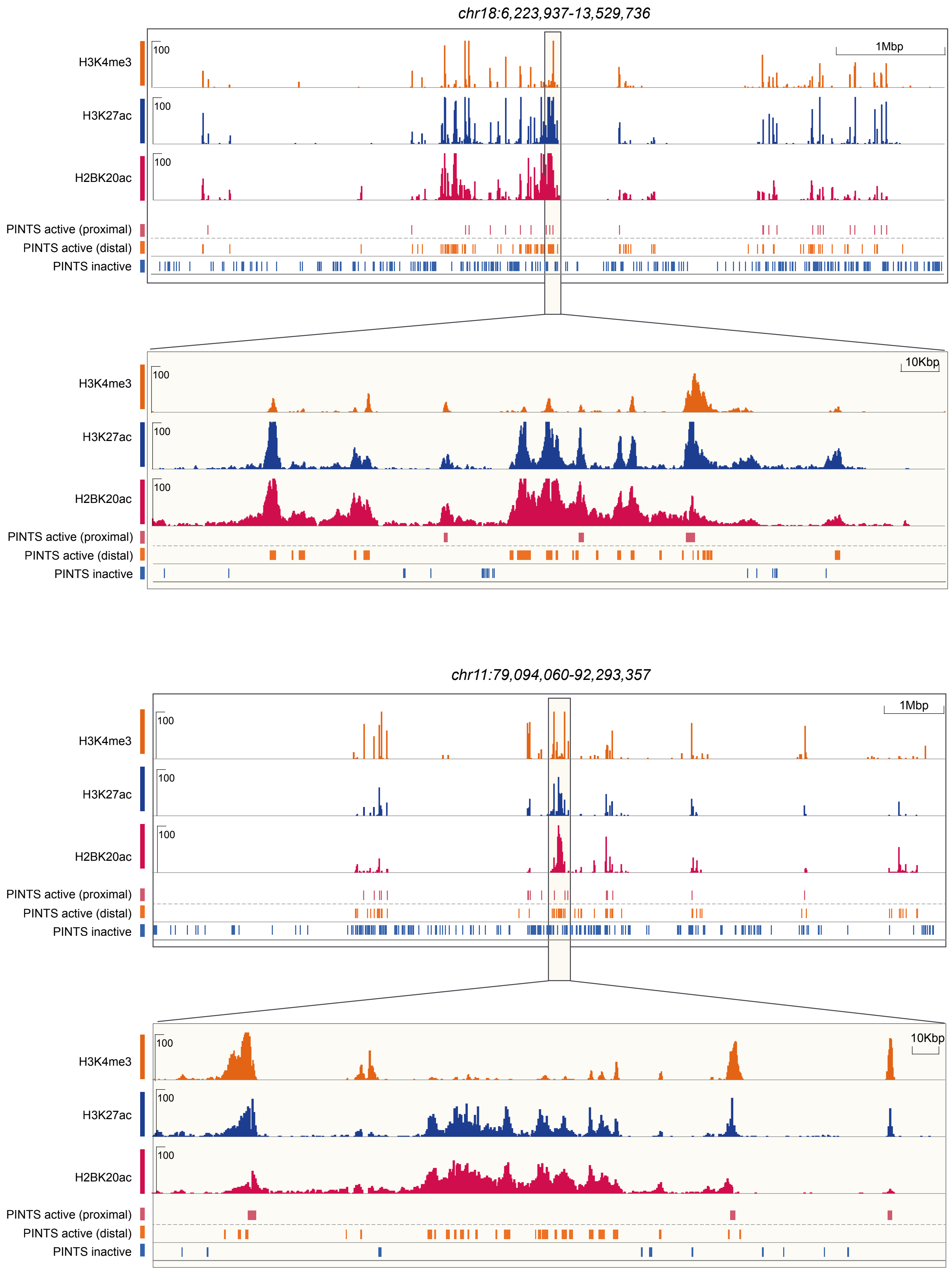

Supplemental Fig. 15. H2BNTac and H3K27ac prominently mark active PINTS regions. Representative genome browser tracks showing the occurrence of H2BNTac and H3K27ac in active and inactive PINTS regions. Two representative regions, and their zoom-in, are shown. The shown regions include hundreds of active and inactive PINTS regions. Of note, a vast majority of active PINTS regions are marked with H2BNTac and H3K27ac, whereas most inactive PINTS regions are either devoid of these acetylation marks or have a much lower level of acetylation. The distinct overlap of acetylation marks with active and inactive PINTS regions is clearer in the zoom-in regions showing a high overlap of acetylation marks with active PINTS regions, and low or lack of acetylation in inactive PINTS regions.

Supplementary Fig. 16

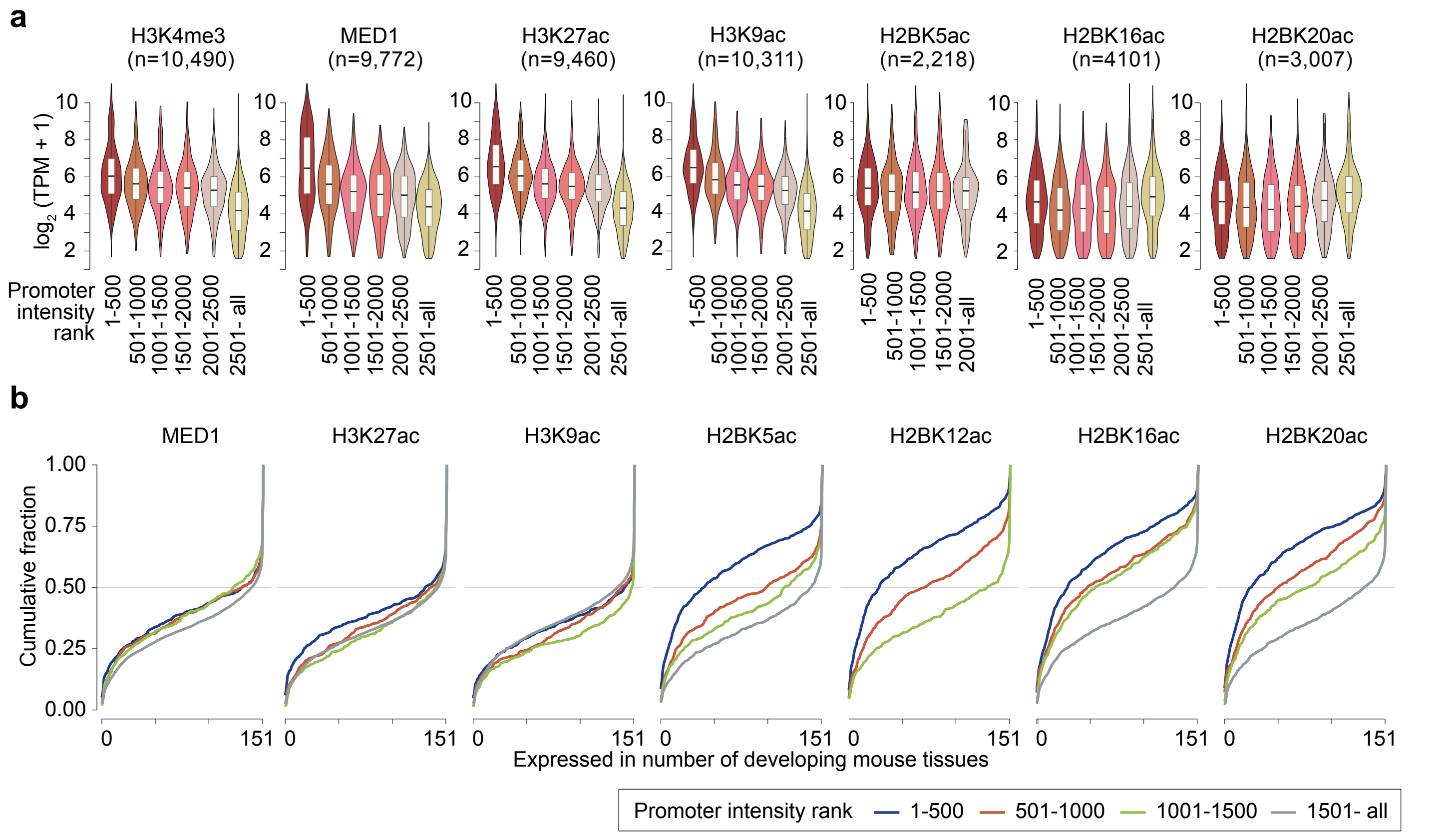

Supplemental Fig. 16. Promoter H2BNTac signal poorly associates with gene expression levels but indicates cell-type-specificity of genes. a, The indicated chromatin marks were rank-ordered based on their ChIP-seq signal intensity in promoters (± 1kb from TSS) of actively transcribed genes in mESC. Shown are gene expressions (TPM +1) within the indicated abundance ranges. **b,** H2BNTac preferentially marks cell-type-specific gene promoters. The indicated chromatin marks were rank-ordered and grouped into different intensity ranges, as described in a. Cell-type-specificity was determined within the indicated abundance ranges. Shown is a cumulative fraction of genes in each group plotted against genes expressed in 151 FANTOM5 CAGE profiles of mouse tissues[24]. **c,** Aggregate plots showing the average p300 ChIP signal in the indicated classes of A-485 regulated gene promoters in mESC. A-485 regulated genes were defined by Narital et al.[21] and are classified as follows: Not changed (N.C., transcription decreased by less than 1.2-fold after A-485 treatment), Slight Down (transcription decreased by ≥1.5-fold after A-485 treatment), Down (transcription decreased by ≥2-fold after A-485 treatment).

Supplementary Fig. 17

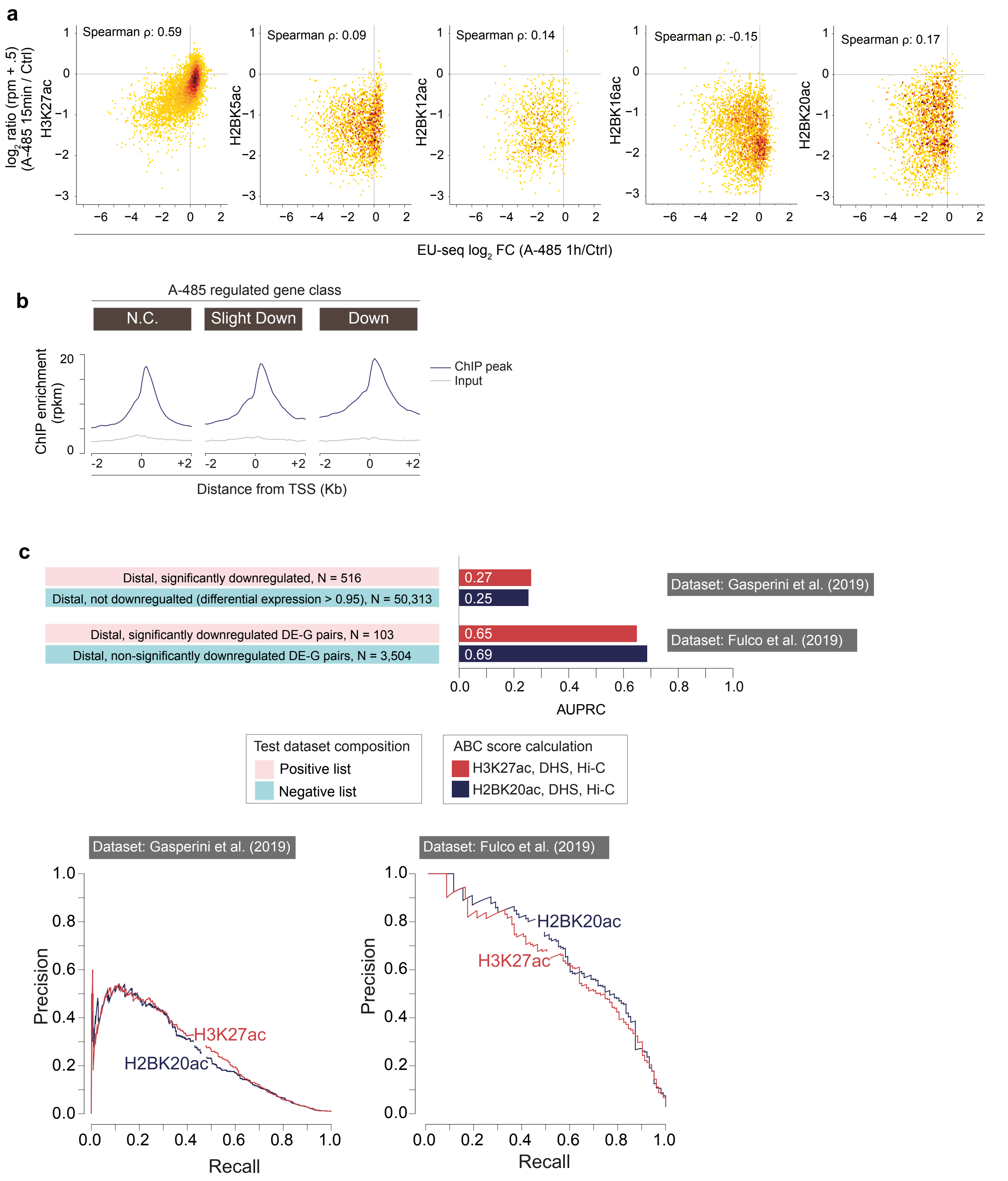

Supplemental Fig. 17. CBP/p300 binding in promoters, association of promoter acetylation with A-485-induced gene regulation, and the performance of H3K27ac and H2BNTac in the context of the ABC model. a, Show is the correlation (Spearman ρ) between A-485-induced changes in promoter-associated H3K27ac or H2BNTac site intensity and A-485-induced gene downregulation. mESC were treated without or with A-485 (15 min) and change in H3K27ac and H2BNTac signal was determined using ChIP-seq. H3K27ac and H2BNTac intensity were quantified within the ±1kb region from TSS. A-485-induced changes in nascent transcription were determined after 1 h of A-485 treatment[21]. **b,** Aggregate plots showing the average p300 ChIP signal in the indicated classes of A-485 regulated gene promoters in mESC. A-485 regulated genes[21] are classified as follows: Not changed (N.C., transcription decreased by less than 1.2-fold after A-485 treatment), Slight Down (transcription decreased by ≥1.5-fold after A-485 treatment), Down (transcription decreased by ≥2-fold after A-485 treatment). **c,** Evaluation of the relative performance of H3K27ac and H2BK20ac for the prediction of CRISPRi-defined enhancer target genes using the ABC model. The dataset of CRISPRi-defined enhancer-gene pairs was obtained from the prior work by Fulco et al. and Gasperini et al[43,46]. For each of the datasets, ABC scores were calculated using the same DHS and Hi-C data as Fulco et al. and in-house generated H3K27ac or H2BK20ac ChIP-seq data. In each dataset, a list of positive and negative genes was defined by applying the indicated criteria. The top panel shows AUPRC values for the indicated datasets, and the bottom panels show precision-recall curves for the respective datasets.

Supplementary Fig. 18

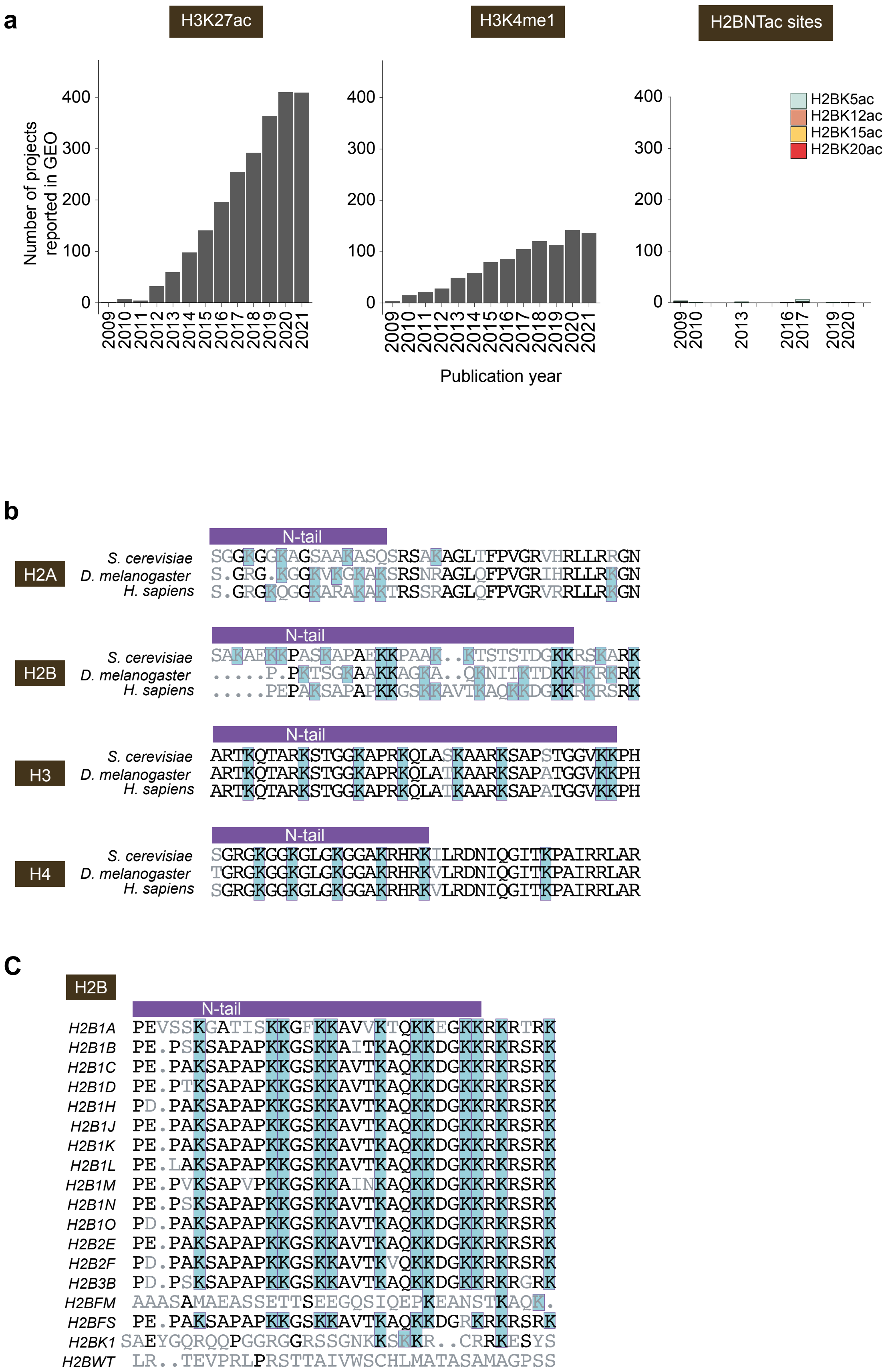

Supplemental Fig. 18. H3K27ac is extensively profiled, whereas H2BNTac is vastly understudied and the H2BNT sequence is poorly conserved among eukaryotes. a, Plotted is the number of H3K27ac, H3K4me1, and H2BNTac site genome-wide mapping projects deposited in the GEO repository from 2009 till 2021. Note that, over the past decade, the number of H3K27ac genome-wide profiling projects reported in GEO has linearly increased year to year. In contrast, the number of deposited H2BNTac projects has not changed over the years, showing that H2BNTac remains understudied. H2BK5ac is profiled more often than other H2BNTac marks, but our analyses indicate that the antibodies used for previously published H2BK5ac ChIP-seq analyses cross-react with H3K27ac (**Supplemental Fig. 5**). Note that the figure shows the number of projects (not the number of individual ChIP-seq experiments) that included genome-wide mapping data for the indicated marks. **b,** The sequence similarity of core histone N-termini among the indicated species. As compared to histones H3 and H4 N-termini, the histone H2B N-terminus shows high sequence divergence. **c,** The sequence similarity of H2BNT among different human histone H2B isoforms.

Supplementary Table 1

Histone mark	Antibody supplier	Cat#	Clone ID	ChIP-qPCR fold-enrichment (Nanog /Hoxa13)
H2BK5ac	Abcam	ab40886	rabbit EP857Y	436.5
H2BK5ac	Cell Signaling Technology	12799S	rabbit D5H1S	10.0
H2BK11ac	ReMAb Biosciences	31-1348-00	rabbit RM456	5.5
H2BK12ac	Abcam	ab40883	rabbit EP858Y	8.4
H2BK12ac	Cell Signaling Technology	9072S	rabbit D7H4	2.9
H2BK15ac	Abcam	ab62335	rabbit EP955Y	1.3
H2BK15ac	Cell Signaling Technology	9083S	rabbit D8H1	1.3
H2BK16ac	Abcam	ab177427	rabbit EPR17598	12.0
H2BK20ac	Abcam	ab177430	rabbit EPR859	35.3
H2BK20ac	Cell Signaling Technology	34156S	rabbit D7O9W	8.2
H3K27ac	Abcam	ab4729	Polyclonal	22.1

Supplementary Table 1. A list of H2BNTac antibodies tested and used for ChIP. Before using for ChIP-seq, the performance of all antibodies was tested using ChIP-qPCR. From chromatin immunoprecipitated samples, fold enrichment of acetylation-positive (*Nanog* enhancer) and acetylation-negative chromatin region (*Hoxa13*) regions were determined using ChIP-qPCR. Antibodies providing >5-fold enrichment were used for ChIP-seq analyses.

Supplementary Table 2

Histone	Peptide sequence
H2B N-terminus (amino acids 1-26) unmodified	H-PEPSKSAPAPKKGSKKAITKAQKKDGGG(K(Biotin))-NH2
H2B N-terminus (amino acids 1-26) with all lysine acetylated	H-PEPSK(ac)SAPAPK(ac)K(ac)GSK(ac)K(ac)AITKAQK(ac)K(ac)DGGG(K(Biotin))-NH2
H2B C-terminus (amino acids 114-125) unmodified	H-GTKAVTKYTSSK-OH
H2B C-terminus (amino acids 114-125) with lysine 120 acetylated	H-GTKAVTK(Ac)YTSSK-OH
H3 (amino acids 10-34) unmodified	H-STGGKAPRKQLATKAARKSAPATGG(K(Biotin))-NH2
H3 (amino acids 10-34) all lysine acetylated	H-STGGK(ac)APRK(ac)QLATK(ac)AARK(ac)SAPATGG(K(Biotin))-NH2

Supplementary Table 2. Sequences of unmodified and acetylated histone peptides that were used for analyzing antibody specificity by quantitative image-based cytometry.

Supplementary Table 3

**Type**	**Name**	**Sequence infromation**	**Purpose**
Plasmid	mHDAC1_C_GFP_dTAG_P2A_Puro.dna	Source data files	Homology arm to generate HDAC1-GFP-FKBP^{12F36V} mouse ES cells
	mHDAC2_C_GFP_dTAG_P2A_Neo.dna	Source data files	Homology arm to generate HDAC2-GFP-FKBP^{12F36V} mouse ES cells
gRNA primer	mHDAC1_C_gd1_F	CACCGTGGTCAAGTTGGCCTGAGCA	PX330 with gRNA to generate HDAC1-GFP-FKBP^{12F36V} mouse ES cells
	mHDAC1_C_gd1_R	AAACTGCTCAGGCCAACTTGACCAC	
	mHDAC1_C_gd2_F	CACCGTGGGGCTGCAGACCTTGCTC	
	mHDAC1_C_gd2_R	AAACGAGCAAGGTCTGCAGCCCCAC	
	mHDAC2_C_gd1_F	CACCGAGTTGGAGAGTCAAATTCAA	PX330 with gRNA to generate HDAC2-GFP-FKBP^{12F36V} mouse ES cells
	mHDAC2_C_gd1_R	AAACTTGAATTTGACTCTCCAACTC	
Primer	mHDAC1_C_scPCR_F1	GGCTCACATACCACCCAAGC	Genotyping primer for HDAC1-GFP-FKBP^{12F36V} mouse ES cells.
	mHDAC1_C_scPCR_R1	AGAGACGGGGAAGAACTTAAACC	
	mHDAC1_C_scPCR_F2	TTGGTCTCTGCTGGCTGACT	
	mHDAC1_C_scPCR_R2	GACGGGGAAGAACTTAAACCAAC	
	mHDAC2_C_scPCR_F1	AGTAAACCTGAGTGCTGACTCATT	Genotyping primer for HDAC2-GFP-FKBP^{12F36V} mouse ES cells.
	mHDAC2_C_scPCR_R1	TCCTGACACAAAGTGACACTGTT	
qPCR primer	Nanog_En_qPCR_F	CTTGGGAGAGAGGGAAAGAAACA	ChIP-qCPR primer for amplifying Nanog enhacer region.
	Nanog_En_qPCR_R	AGTCAGGACCTCACTATGTCAGA	
	Hoxa13.intron_En_qPCR_F	CACCAAATTGTCCCTGATGGTTC	ChIP-qCPR primer for amplifying Hoxa13 intron region.
	Hoxa13.intron_En_qPCR_R	ACACTTCTTTCTGTAGAGCTCGG	

Supplementary Table 3. Oligonucleotide sequences that were used for generating HDAC1/2-GFP-FKBP12^{F36V} mESC and for ChIP-qPCR.

Source Data Fig.1

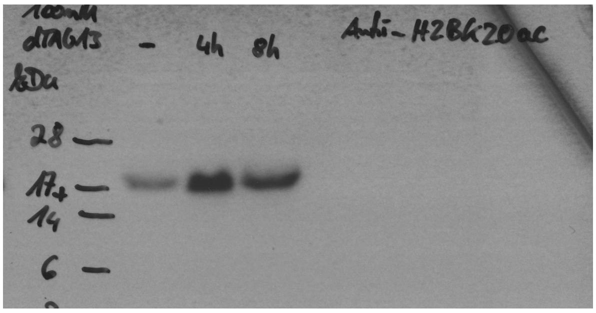

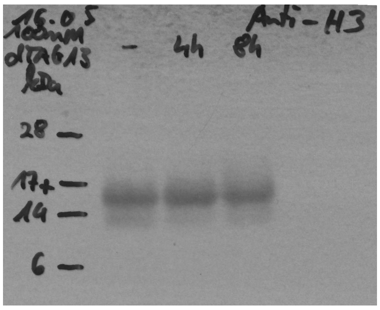

Source data Fig1. Uncropped scans of immunobolot films shown in Supplemental Figure 12b. Iimmunoblotted for H2BK20ac (top) and total histone H3 (bottom).
