# Supplementary material for: Acetylation of histone H2B marks active enhancers and predicts CBP/p300 target genes
Source: Nat Genet. 2023 Apr 6;55(4):679–92. doi: 10.1038/s41588-023-01348-4 (PMC10101849; doi:10.1038/s41588-023-01348-4)
Supplement: Supplementary file 2 — Reporting Summary [file 41588_2023_1348_MOESM2_ESM.pdf]

nature portfolio

Corresponding author(s): Chunaram Choudhary

Last updated by author(s): 27/01/23

Reporting Summary

Nature Portfolio wishes to improve the reproducibility of the work that we publish. This form provides structure for consistency and transparency in reporting. For further information on Nature Portfolio policies, see our Editorial Policies and the Editorial Policy Checklist.

Statistics

For all statistical analyses, confirm that the following items are present in the figure legend, table legend, main text, or Methods section.

n/a	Confirmed	
☐	☒	The exact sample size (*n*) for each experimental group/condition, given as a discrete number and unit of measurement
☐	☒	A statement on whether measurements were taken from distinct samples or whether the same sample was measured repeatedly
☐	☒	The statistical test(s) used AND whether they are one- or two-sided<br>*Only common tests should be described solely by name; describe more complex techniques in the Methods section.*
☐	☒	A description of all covariates tested
☐	☒	A description of any assumptions or corrections, such as tests of normality and adjustment for multiple comparisons
☐	☒	A full description of the statistical parameters including central tendency (e.g. means) or other basic estimates (e.g. regression coefficient) AND variation (e.g. standard deviation) or associated estimates of uncertainty (e.g. confidence intervals)
☐	☒	For null hypothesis testing, the test statistic (e.g. *F*, *t*, *r*) with confidence intervals, effect sizes, degrees of freedom and *P* value noted<br>*Give P values as exact values whenever suitable.*
☒	☐	For Bayesian analysis, information on the choice of priors and Markov chain Monte Carlo settings
☒	☐	For hierarchical and complex designs, identification of the appropriate level for tests and full reporting of outcomes
☐	☒	Estimates of effect sizes (e.g. Cohen's *d*, Pearson's *r*), indicating how they were calculated

Our web collection on statistics for biologists contains articles on many of the points above.

Software and code

Policy information about availability of computer code

Data collection	The statistics of ChIP-seq projects in GEO: reutils (version 0.2.3).
Data analysis	Sequencing analysis: Cutadapt(4.2), FastQC(0.11.5), Bedtools(2.23), HTseq(0.11.1), BWA(0.7.10), samtools(1.4), LanceOtron(1.0.8), R(4.1.1), PRROC (1.3), ChIPseeker (1.28.3), GenomicAlignments (1.8.4), GenomicRanges (1.44.0), stats (3.6.2), ggplot2(3.3.5), DESeq(1.32.0), ChIPseeker(l.28.3), msa(1.24.0), deeptools(3.5.0), JUICER (1.6), IGV(2.10.3), ChromHMM(1.22), ABC model pipeline (https://github.com/broadinstitute/ABC-Enhancer-Gene-Prediction). PRROC (1.3), ChIPseeker (1.28.3), GenomicAlignments (1.8.4), GenomicRanges (1.44.0).<br><br>Microscopic analysis: Harmony High-Content Imaging and Analysis (4.9), Olympus ScanR Image Analysis(2.8).

For manuscripts utilizing custom algorithms or software that are central to the research but not yet described in published literature, software must be made available to editors and reviewers. We strongly encourage code deposition in a community repository (e.g. GitHub). See the Nature Portfolio guidelines for submitting code & software for further information.

Data

Policy information about availability of data

All manuscripts must include a data availability statement. This statement should provide the following information, where applicable:

- Accession codes, unique identifiers, or web links for publicly available datasets
- A description of any restrictions on data availability
- For clinical datasets or third party data, please ensure that the statement adheres to our policy

This project's sequencing raw data, processed peak regions, and gene tracks are available on the National Center for Biotechnology Information Gene Expression Omnibus (GEO) under accession number GSE186349.

Additionally, the following datasets are downloaded and analyzed.
Gene annotation for human and mice: (https://www.gencodegenes.org)
Gene expression profiles based on promoter transcripts: FANTOM5 CAGE dataset, array express (http://www.ebi.ac.uk/arrayexpress/)
Encode DAC Blacklisted Regions (https://www.encodeproject.org/annotations/ENCSR636HFF/)
Super enhancer regions: dbSuper (http://bioinfo.au.tsinghua.edu.cn/dbsuper/)
Genome states annotation: ESC 18 chromHMM states (https://www.encodeproject.org/search/?searchTerm=ChromHMM+Zhiping+Weng)
K562 25 chromHMM states (http://hgdownload.cse.ucsc.edu/goldenpath/hg19/encodeDCC/wgEncodeAwgSegmentation)
Transcription-supported enhancers and promoters: PINTS elements (https://pints.yulab.org/summary_stats)
Plasmid-based assay-supported enhancers: ESC STARR-seq peaks (Supplemental data section of Peng et.al (PMID: 32912294))
Plasmid-based assay-supported autonomous promoters: K562 SuRE peaks (Supplementary section of van Arensbergen et al. (PMID: 28024146))
Regulatory interactions between enhancers and genes based on CRISPRi perturbations: K562 CRISPRi screen (Supplemental data section of Fulco et.al (41588_2019_538_MOESM3_ESM.xlsx, PMID: 31784727), Supplemental data of Gasperini et al. (GSE120861_all_deg_results.at_scale.txt, PMID: 30612741)

We re-analyzed the following publicly available sequencing datasets.
ESC H3K27ac (GSE135562, GSE160890), K562 H3K9ac (GSE29611), ESC H3K4me1 (GSE146324), ESC NANOG (GSE146324), ESC OCT4 (GSE146324), ESC p300 (GSE146324), ESC H3K36me3 (GSE118785), ESC H3K9me3 (GSE90895), ESC CTCF(GSE178982), K562 H3K4me3 (GSE163049), and K562 H3K4me1 (GSE29611, GSE31755). ESC RNA-seq (GSE146324). ESC Hi-C (GSE118911), K562 Hi-C (GSE63525), ESC Micro-C (GSE130275), ESC DNase-Seq (GSE37074), K562 DNase-Seq (GSE29692)

Field-specific reporting

Please select the one below that is the best fit for your research. If you are not sure, read the appropriate sections before making your selection.

☒ Life sciences ☐ Behavioural & social sciences ☐ Ecological, evolutionary & environmental sciences

For a reference copy of the document with all sections, see nature.com/documents/nr-reporting-summary-flat.pdf

Life sciences study design

All studies must disclose on these points even when the disclosure is negative.

Sample size	Image-based cytometry: 3000 randomly sampled cells from two independent experiments were analyzed.
Data exclusions	Image-based cytometry: Cells that did not show 2C-4C DNA based on total and mean DAPI intensities were excluded.
Replication	We generally performed at least two biologically independent experiments per antigen and conditions. For the following ChIP-seq experiments, single replicate was performed: H2BK5ac(ab40866), H2BK11ac, H3K9ac, H3K4me3, H3K27me3 ChIP in ESC ; H3K27ac ChIP in K562 cells on control condition, or A-485 5 min treatment, and H2BK20ac ChIP in ESC on NVP-2 120 min treatment condition. Immunoblotting, flow cytometry experiments were repeated at least twice independently. All attempts to replicate the results were successful.
Randomization	Samples were grouped according to antigen and experimental condition, and each group was analyzed individually. A-485 or NVP-2 treated samples were paired with the corresponding controls.
Blinding	The investigators were not blinded because the study did not involve any clinical samples. All experiments were done in cell lines, and blinding was not necessary as the data were mainly generated by digital reading. For all samples, data analysis was performed using the same pipeline.

Reporting for specific materials, systems and methods

We require information from authors about some types of materials, experimental systems and methods used in many studies. Here, indicate whether each material, system or method listed is relevant to your study. If you are not sure if a list item applies to your research, read the appropriate section before selecting a response.

Materials & experimental systems

n/a	Involved in the study	
☐	☒	Antibodies
☐	☒	Eukaryotic cell lines
☒	☐	Palaeontology and archaeology
☒	☐	Animals and other organisms
☒	☐	Human research participants
☒	☐	Clinical data
☒	☐	Dual use research of concern

Methods

n/a	Involved in the study	
☐	☒	ChIP-seq
☒	☐	Flow cytometry
☒	☐	MRI-based neuroimaging

Antibodies

Antibodies used

ChIP-seq
Antibody Antibody supplier Cat# Clone ID/ Lot volume of antibody for each ChIP experiment (ug or ul)
anti-H2BK5ac Abcam ab40886 rabbit EP857Y 2ug
anti-H2BK5ac Cell Signaling Technology 12799S rabbit D5H1S 10ul
anti-H2BK11ac ReMAb Biosciences 31-1348-00 rabbit RM456 3ug
anti-H2BK12ac Abcam ab40883 rabbit EP858Y 2ug
anti-H2BK16ac Abcam ab177427 rabbit EPR17598 2ug
anti-H2BK20ac Abcam ab177430 rabbit EPR859 3.5ug
anti-H2BK20ac Cell Signaling Technology 34156 rabbit D7O9W 10ul
anti-H3K27ac Abcam ab4729 rabbit 2ug
anti-H3K9ac CST #9671 rabbit 10ul
anti-Med1 Millipore 17-10530 rabbit 3.45ug
anti-H3K4me3 Millipore 04-745 rabbit MC315 5ul
anti-H3K27me3 CST #9733 rabbit C36B11 5ul

Immunostaining
Antibody Antibody supplier Cat# Clone ID/ Lot Dilution
anti-H2BK5ac Active motif 39124 rabbit 229 1:1000
anti-H2BK5ac abcam ab40886 rabbit GR111987-5 1:1000
anti-H2BK5ac Cell Signaling Technologies 12799S rabbit 09/2020 lot 1 1:1000
anti-H2BK20ac Cell Signaling Technologies 34156 rabbit 04/2020 1:1000
anti-H2BK20ac abcam ab177430 rabbit GR3196303-2 1:1000
anti-H2BK120ac Upstate 07-564 rabbit 26872 1:1000
anti-H2BK120ac Active motif 39119 rabbit 1008001 1:1000
anti-H3K27ac abcam ab177178 rabbit GR202987-5 1:1000
anti-GFP abcam ab290 rabbit 70828032ab 1:250
Alexa Fluor A488 anti-rabbit Thermo Fisher A11034 rabbit 2110499 1:500, or 1:1000

Western blotting
Antibody Antibody supplier Cat# Clone ID/ Lot Dilution
Peroxidase AffiniPure F(ab')$_2$ Fragment Goat anti-Rabbit IgG (H+L) Jackson Immuno Research 111-036-045 goat 107341 1:5000
anti-H2BK20ac Cell Signaling Technology 34156 rabbit D7O9W 1:1000
anti-H3 Cell Signaling Technologies 4499 rabbit 9 1:1000

Validation

Anti-H2BK5ac Abcam ab40886: This antibody cross-react with H3K27ac as shown in this manuscript. The manufacturer has also added this information in the datasheet for this antibody (https://www.abcam.com/histone-h2b-acetyl-k5-antibody-ep857y-chip-grade-ab40886.html).

Anti-H2BK5ac Cell Signaling Technology 12799S: According to the manufacturer's website, Acetyl-Histone H2B (Lys5) (D5H1S) XP® Rabbit mAb recognizes endogenous levels of histone H2B only when acetylated at Lys5. There is no cross-reactivity between this antibody and other acetylated histones. (https://www.cellsignal.com/products/primary-antibodies/acetyl-histone-h2b-lys5-d5h1s-xp-rabbit-mab/12799)

Anti-H2BK5ac Active motif 39124: According to the antibody datasheet, this antibody was validated by modENCODE and NIH Roadmap Epigenomics Mapping Consortiums. Our results indicate that this antibody also cross-reacts with H3K27ac.

anti-H2BK11ac ReMAb Biosciences 31-1348-00: According to the manufacturer's page, this antibody reacts to Histone H2B acetylated at Lysine11 (K11ac). No cross-reactivity with other acetylated Lysines in histones. (https://www.revmab.com/index.php/product/anti-acetyl-histone-h2b-lys11-rabbit-monoclonal-antibody-clone-rm456/)

anti-H2BK12ac Abcam ab40883: According to the manufacturer's page, this antibody only detects Histone H2B acetylated on Lysine 12. (https://www.abcam.com/histone-h2b-acetyl-k12-antibody-ep858y-chip-grade-ab40883.html)

anti-H2BK16ac Abcam ab177427: According to the manufacturer, this antibody selectively recognize H2BK16ac. The specificity of ab177430 was tested by the manufacturer in a Peptide array against 501 different modified and unmodified histone peptides; each peptide is printed on the array at six concentrations (https://www.abcam.com/histone-h2b-acetyl-k16-antibody-epr17598-chip-grade-ab177427.html).

anti-H2BK20ac Abcam ab177430: According to the manufacturer, this antibody selectively recognize H2BK20ac. The specificity of ab177430 was tested in a Peptide array against 501 different modified and unmodified histone peptides; each peptide is printed on the array at six concentrations (https://www.abcam.com/histone-h2b-acetyl-k20-antibody-epr859-chip-grade-ab177430.html)

anti-H2BK20ac Cell Signaling Technology 34156: According to the manufacturer's page, Acetyl-Histone H2B (Lys20) (D7O9W) Rabbit mAb recognizes endogenous levels of histone H2B protein when acetylated at Lys20. This antibody shows very slight cross-reactivity with histone H2B acetylated at Lys12.(https://www.cellsignal.com/products/primary-antibodies/acetyl-histone-h2b-lys20-d7o9w-rabbit-mab/34156)

anti-H3K27ac Abcam ab4729: According to the manufacturer, all batches of ab4729 are tested in Peptide Array against peptides to different Histone H3 modifications. Results show strong binding to Histone H3K27ac peptide.

anti-H3K27ac Abcam ab177178: According to the manufacturer, ab177178 was tested in a Peptide array against 501 modified and

unmodified histone peptides. Results show strong binding to Histone H3K27ac peptide.

anti-H2BK120ac Upstate 07-564: According to the manufacturer, this antibody recognizes H2BK120ac. Our results indicate that this antibody also cross-reacts with H2BK20ac.

anti-H2BK120ac Active motif 39119: According to the manufacturer, this antibody recognizes H2BK120ac. Our results indicate that this antibody also cross-reacts with H2BK20ac.

anti-H3 Cell Signaling Technologies 4499: The antibody is validated by the manufacturer, and according to the antibody datasheet, this antibody has been used in >1000 papers.

anti-GFP abcam ab290: The antibody is validated by the manufacturer, and according to the antibody datasheet, this antibody has been used in >2500papers.

H3K27me3 CST #9733: According to the manufacturer's page, the antibody does not cross-react with non-methylated, mono-methylated, or di-methylated Lys27. In addition, the antibody does not cross-react with mono-methylated, di-methylated, or tri-methylated histone H3 at Lys4, Lys9, Lys36, or Histone H4 at Lys20 (https://www.cellsignal.com/products/primary-antibodies/tri-methyl-histone-h3-lys27-c36b11-rabbit-mab/9733).

H3K9ac CST 9671: According to the manufacturer's page, the acetyl-Histone H3 (Lys9) Antibody detects endogenous levels of Histone H3 only when acetylated at lysine 9. It does not cross-react with phosphorylated histone H3 (https://www.cellsignal.com/product/productDetail.jsp?productId=9671).

Med1 Millipore 17-10530: The manufacturer validates this antibody using ChIP-qPCR (https://www.merckmillipore.com/DK/en/product/ChIPAb-MED1-Antibody-ChIP-Validated-Antibody-and-Primer-Set,MM_NF-17-10530?ReferrerURL=https%3A%2F%2Fwww.google.com%2F#anchor_keySpecTable).

Eukaryotic cell lines

Policy information about cell lines

Cell line source(s)

K562: source ATCC, cat# CCL 243
hTERT RPE-1: source ATCC (Cat# CRL-4000)
Mouse ESC were generously provided by the SyBOSS consortium partners (Francis Stewart, Austin Smith, Bill Skarnes).

Authentication

All human cell lines were authenticated by Eurofins Genomics using PCR-single-locus-technology. Identity of ESC was confirmed by the expression of cell-type-specific markers by RNA-seq.

Mycoplasma contamination

Cell lines were tested for mycoplasma contamination every 2-3 months, and were confirmed mycoplasma negative.

Commonly misidentified lines
(See ICLAC register)

None of the used cell lines are listed in the commonly misidentified lines.

ChIP-seq

Data deposition

☒ Confirm that both raw and final processed data have been deposited in a public database such as GEO.

☒ Confirm that you have deposited or provided access to graph files (e.g. BED files) for the called peaks.

Data access links
May remain private before publication.

GEO:GSE186349

Files in database submission

ChIP-seq:
ESC_Ctrl_TC0_H2BK5ac.ab40886_CC8_1.fastq.gz
ESC_A485_10uM_TC15_H2BK5ac.ab40886_CC8_1.fastq.gz
ESC_Ctrl_TC0_H2BK5ac.CST12799_CC1_1.fastq.gz
ESC_Ctrl_TC0_H2BK5ac.CST12799_CC1_2.fastq.gz
ESC_Ctrl_TC0_H2BK5ac.CST12799_CC15_1.fastq.gz
ESC_Ctrl_TC0_H2BK5ac.CST12799_CC11_1.fastq.gz
ESC_A485_10uM_TC15_H2BK5ac.CST12799_CC1_1.fastq.gz
ESC_A485_10uM_TC15_H2BK5ac.CST12799_CC1_2.fastq.gz
ESC_Ctrl_TC0_H2BK12ac.ab40883_CC8_1.fastq.gz
ESC_Ctrl_TC0_H2BK12ac.ab40883_CC15_1.fastq.gz
ESC_A485_10uM_TC15_H2BK12ac.ab40883_CC8_1.fastq.gz
ESC_A485_10uM_TC15_H2BK12ac.ab40883_CC15_1.fastq.gz
ESC_Ctrl_TC0_H2BK16ac.ab177427_CC8_1.fastq.gz
ESC_Ctrl_TC0_H2BK16ac.ab177427_CC15_1.fastq.gz
ESC_A485_10uM_TC15_H2BK16ac.ab177427_CC8_1.fastq.gz
ESC_A485_10uM_TC15_H2BK16ac.ab177427_CC15_1.fastq.gz
ESC_Ctrl_TC0_H2BK20ac.ab177430_CC8_1.fastq.gz

ESC_Ctrl_TC0_H2BK20ac.ab177430_CC15_1.fastq.gz
ESC_Ctrl_TC0_H2BK20ac.ab177430_CC19_1.fastq.gz
ESC_A485_10uM_TC15_H2BK20ac.ab177430_CC8_1.fastq.gz
ESC_A485_10uM_TC15_H2BK20ac.ab177430_CC15_1.fastq.gz
ESC_Ctrl_TC0_H2BK20ac.CST34156_CC15_1.fastq.gz
ESC_A485_10uM_TC15_H2BK20ac.CST34156_CC15_1.fastq.gz
ESC_Ctrl_TC0_Med1_dfix_Spike.HEK293_CC10_1.fastq.gz
ESC_Ctrl_TC0_Med1_dfix_Spike.HEK293_CC12_1.fastq.gz
ESC_Ctrl_TC0_H2BK20ac.CST34156_CC27_1.fastq.gz
ESC_NVP2_100nM_TC120_H2BK20ac.CST34156_CC27_1.fastq.gz
ESC_Ctrl_TC0_H2BK11ac.RM456_CC20_1.fastq.gz
ESC_Ctrl_TC0_H3K4me3_CC10_1.fastq.gz
ESC_Ctrl_TC0_H3K9ac_CC14_1.fastq.gz
ESC_Ctrl_TC0_H3K27me3_CC8_1.fastq.gz
K562_Ctrl_TC0_H2BK20ac.ab177430_CC16_1.fastq.gz
K562_Ctrl_TC0_H2BK20ac.CST34156_CC20_1.fastq.gz
K562_Ctrl_TC0_H3K27ac.ab4729_CC10_1.fastq.gz

Genome browser session
(e.g. UCSC)

NA

Methodology

Replicates

Single ChIP-seq replicate was performed in the following samples: H2BK5ac(ab40866), H2BK11ac, H3K9ac, H3K4me3, H3K27me3 in ESC and to map H3K27ac in K562 cells on control condition. A-485 5 min treatment and H2BK20ac ChIP in ESC on NVP-2 120 min treatment condition. Other ChIP-seq experiments were in two or more biological replicates.

Sequencing depth

ChIP-seq:
ESC_Ctrl_TC0_H2BK5ac.ab40886_CC8_1.fastq.gz total: 34589169 unique: 25018109 75bp single-end
ESC_A485_10uM_TC15_H2BK5ac.ab40886_CC8_1.fastq.gz total: 34730315 unique: 25082513 75bp single-end
ESC_Ctrl_TC0_H2BK5ac.CST12799_CC1_1.fastq.gz total: 54917433 unique: 38503438 75bp single-end
ESC_Ctrl_TC0_H2BK5ac.CST12799_CC1_2.fastq.gz total: 56102173 unique: 39752879 75bp single-end
ESC_Ctrl_TC0_H2BK5ac.CST12799_CC15_1.fastq.gz total: 36220680 unique: 25711848 75bp single-end
ESC_Ctrl_TC0_H2BK5ac.CST12799_CC11_1.fastq.gz total: 31431198 unique: 20844959 75bp single-end
ESC_A485_10uM_TC15_H2BK5ac.CST12799_CC1_1.fastq.gz total: 59354741 unique: 41082159 75bp single-end
ESC_A485_10uM_TC15_H2BK5ac.CST12799_CC1_2.fastq.gz total: 56323325 unique: 39203285 75bp single-end
ESC_Ctrl_TC0_H2BK12ac.ab40883_CC8_1.fastq.gz total: 34085582 unique: 25128054 75bp single-end
ESC_Ctrl_TC0_H2BK12ac.ab40883_CC15_1.fastq.gz total: 31747589 unique: 23745357 75bp single-end
ESC_A485_10uM_TC15_H2BK12ac.ab40883_CC8_1.fastq.gz total: 31094097 unique: 22239318 75bp single-end
ESC_A485_10uM_TC15_H2BK12ac.ab40883_CC15_1.fastq.gz total: 33282776 unique: 24517700 75bp single-end
ESC_Ctrl_TC0_H2BK16ac.ab177427_CC8_1.fastq.gz total: 33306788 unique: 24418077 75bp single-end
ESC_Ctrl_TC0_H2BK16ac.ab177427_CC15_1.fastq.gz total: 28793790 unique: 21965837 75bp single-end
ESC_A485_10uM_TC15_H2BK16ac.ab177427_CC8_1.fastq.gz total: 32313984 unique: 23562769 75bp single-end
ESC_A485_10uM_TC15_H2BK16ac.ab177427_CC15_1.fastq.gz total: 33637842 unique: 25096858 75bp single-end
ESC_Ctrl_TC0_H2BK20ac.ab177430_CC8_1.fastq.gz total: 33346076 unique: 24429920 75bp single-end
ESC_Ctrl_TC0_H2BK20ac.ab177430_CC15_1.fastq.gz total: 31915477 unique: 24135271 75bp single-end
ESC_Ctrl_TC0_H2BK20ac.ab177430_CC19_1.fastq.gz total: 36479905 unique: 24698633 75bp single-end
ESC_A485_10uM_TC15_H2BK20ac.ab177430_CC8_1.fastq.gz total: 31152039 unique: 22319165 75bp single-end
ESC_A485_10uM_TC15_H2BK20ac.ab177430_CC15_1.fastq.gz total: 28526891 unique: 21228677 75bp single-end
ESC_Ctrl_TC0_H2BK20ac.CST34156_CC15_1.fastq.gz total: 22737115 unique: 17146650 75bp single-end
ESC_A485_10uM_TC15_H2BK20ac.CST34156_CC15_1.fastq.gz total: 30444009 unique: 22533354 75bp single-end
ESC_Ctrl_TC0_Med1_dfix_Spike.HEK293_CC10_1.fastq.gz total: 28787747 unique: 14300032 75bp single-end
ESC_Ctrl_TC0_Med1_dfix_Spike.HEK293_CC12_1.fastq.gz total: 34914638 unique: 10244526 75bp single-end
ESC_Ctrl_TC0_H2BK20ac.CST34156_CC27_1.fastq.gz total: 37810933 unique: 30406203 75bp single-end
ESC_NVP2_100nM_TC120_H2BK20ac.CST34156_CC27_1.fastq.gz total: 40411831 unique: 32516846 75bp single-end
ESC_Ctrl_TC0_H2BK11ac.RM456_CC20_1.fastq.gz total: 30338672 unique: 23964425 75bp single-end
ESC_Ctrl_TC0_H3K9ac_CC14_1.fastq.gz total: 33695764 unique: 25289334 75bp single-end
ESC_Ctrl_TC0_H3K4me3_CC10_1.fastq.gz total: 28251742 unique: 19797653 75bp single-end
ESC_Ctrl_TC0_H3K27me3_CC8_1.fastq.gz total: 34075541 unique: 26440139 75bp single-end
K562_Ctrl_TC0_H2BK20ac.ab177430_CC16_1.fastq.gz total: 32428667 unique: 26093021 75bp single-end
K562_Ctrl_TC0_H2BK20ac.CST34156_CC20_1.fastq.gz total: 42894132 unique: 35626866 75bp single-end
K562_Ctrl_TC0_H3K27ac.ab4729_CC10_1.fastq.gz total: 27374349 unique: 21414657 75bp single-end

Antibodies

anti-H2BK5ac: abcam, ab40886, rabbit EP857Y
anti-H2BK5ac: Cell Signaling Technology, cat# CST12799, clone# rabbit D5H1S
anti-H2BK12ac: abcam cat# ab40883, clone# rabbit EP858Y
anti-H2BK16ac: abcam cat# ab177427, clone# rabbit EPR17598
anti-H2BK20ac: abcam cat# ab177430, clone# rabbit EPR859
anti-H2BK20ac: Cell Signaling Technology cat# 34156, clone# rabbit D7O9W
anti-H3K27ac: abcam cat# ab4729, clone# Rabbit polyclonal
anti-H3K9ac: Cell Signaling Technology, cat# 9671, clone# rabbit polyclonal
anti-H2BK11ac: RevMAb Bioscience, cat# 31-1348-00, clone# rabbit RM456

anti-H3K27me3: CST #9733, rabbit C36B11
anti-H3K4me3: Millipore 04-745, rabbit MC315
anti-Med1: Millipore 17-10530, clone# rabbit polyclonal

Peak calling parameters	Reads were mapped to the reference genome by using bwa aln with default parameters (BWA version 0.7.10). Multi-mapped reads, duplicated reads, or reads with more than three mismatches were removed by samtools. Reads mapped to the DAC Blacklisted Regions (https://www.encodeproject.org/annotations/ENCSR636HFF/) were omitted from the downstream analysis. Peak regions were called using LanceOtron(1.0.8) with default model (wide-and-deep_jan-2021) (doi: https://doi.org/10.1101/2021.01.25.428108). The peaks proximal within 2Kbp are merged using Bedtools, and poorly enriched peaks of maximum peak height < 8 reads mapped per million (rpm) were omitted.
Data quality	FastQC was used for quality check of sequencing reads.
Software	Sequencing analysis: Cutadapt(4.2), FastQC(0.11.5), Bedtools(2.23), HTseq(0.11.1), BWA(0.7.10), samtools(1.4), LanceOtron(1.0.8), R(4.1.1), PRROC (1.3), ChIPseeker (1.28.3), GenomicAlignments (1.8.4), GenomicRanges (1.44.0), stats (3.6.2), ggplot2(3.3.5), DESeq(1.32.0), ChIPseeker(l.28.3), msa(1.24.0), deeptools(3.5.0), JUICER (1.6), IGV(2.10.3), ChromHMM(1.22), ABC model pipeline (https://github.com/broadinstitute/ABC-Enhancer-Gene-Prediction). PRROC (1.3), ChIPseeker (1.28.3), GenomicAlignments (1.8.4), GenomicRanges (1.44.0).
